# Cohort Trajectories by Age and Gender for Informal Caregiving in Europe Adjusted for Sociodemographic Changes, 2004 and 2015

**DOI:** 10.1093/geronb/gbad011

**Published:** 2023-01-23

**Authors:** Ricardo Rodrigues, Johan Rehnberg, Cassandra Simmons, Stefania Ilinca, Eszter Zólyomi, Afshin Vafaei, Selma Kadi, Janet Jull, Susan P Phillips, Stefan Fors

**Affiliations:** ISEG Lisbon School of Economics and Management, SOCIUS—Research Centre in Economic and Organizational Sociology/CSG—Research in Social Sciences and Management, Lisboa, Portugal; European Centre for Social Welfare Policy and Research, Vienna, Austria; Aging Research Center, Karolinska Institutet & Stockholm University, Stockholm, Sweden; European Centre for Social Welfare Policy and Research, Vienna, Austria; World Health Organization, Regional Office for Europe, Copenhagen, Denmark; European Centre for Social Welfare Policy and Research, Vienna, Austria; Department of Public Health Sciences & Department of Family Medicine, Queen’s University, Kingston, Ontario, Canada; European Centre for Social Welfare Policy and Research, Vienna, Austria; School of Rehabilitation Therapy, Queen’s University, Kingston, Ontario, Canada; Department of Public Health Sciences & Department of Family Medicine, Queen’s University, Kingston, Ontario, Canada; Aging Research Center, Karolinska Institutet, Solna, Sweden; Department of Public Health Sciences, Centre for Epidemiology and Community Medicine, Stockholm, Sweden

**Keywords:** Cohort analysis, Inequalities, Multilevel models

## Abstract

**Objectives:**

We present a dynamic view of gender patterns in informal caregiving across Europe in a context of sociodemographic transformations. We aim to answer the following research questions: (a) has the gender gap in informal caregiving changed; (b) if so, is this due to changes among women and/or men; and (c) has the gender care gap changed differently across care regimes?

**Methods:**

Multilevel growth curve models are applied to gendered trajectories of informal caregiving of a panel sample of 50+ Europeans, grouped into 5-year cohorts and followed across 5 waves of the Survey of Health, Ageing and Retirement in Europe survey, stratified by sex and adjusted for several covariates.

**Results:**

For men in cohorts born more recently, there is a decrease in the prevalence of informal care outside the household, whereas cohort trajectories for women are mostly stable. Prevalence of care inside the household has increased for later-born cohorts for all without discernible changes to the gender care gap. Gender care gaps overall widened among later-born cohorts in the Continental cluster, whereas they remained constant in Southern Europe, and narrowed in the Nordic cluster.

**Discussion:**

We discuss the cohort effects found in the context of gender differences in employment and care around retirement age, as well as possible demographic explanations for these. The shift from care outside to inside the household, where it mostly consists of spousal care, may require different policies to support carers, whose age profile and possible care burden seem to be increasing.

Presently, informal caregivers meet the majority of care needs of adults with disability or functional limitations in Europe (OECD, 2011). As with care provided to young children earlier in the life course, caregiving to spouses, parents, other relatives, or friends is characterized by a pervasive gender care gap, defined as the difference in the probability to provide care between women and men (Marks, 1996; Ophir, 2022; Ophir & Polos, 2022; Patterson & Margolis, 2019). This marks caregiving as a key feature of the gendered life course. Over the past decades, however, a number of sociodemographic transformations have occurred in the life course for women and men, particularly in the denominated “encore adulthood” (Moen, 2016) and in older adult years, raising the prospect of changes in the overall patterns of caregiving and, with it, shifts in the gender care gap (Agree & Glaser, 2009; Grigoryeva, 2017; OECD, 2011). Investigating the evolution of the gender care gap is relevant from a gender equality perspective, but also to ascertain whether informal caregiving is still available for later-born cohorts of adults and particularly older people.

Among people aged 50 and older, the prevalence of caregiving among both women and men is higher in the years leading up to retirement age, as is the gender care gap (Dahlberg et al., 2007; Ophir & Polos, 2022). The gender care gap in this life stage—mostly referring to caregiving to adults residing outside the carer’s own household—has been associated with differences in the employment and social roles experienced throughout the life course of women and men (Glauber, 2017). Full-time employment has a negative effect on caregiving provided to non-cohabitating older parents, as well as spouses, for both women and men (Glauber, 2017; He & McHenry, 2016; Henz, 2010; Sarkisian, & Gerstel, 2004), but as men remain in full-time employment till retirement age, they have, until now, been less likely to take on caregiving roles in later stages of their lives. As a result, there are deeply gendered life trajectories in paid and unpaid work (i.e., care) not only around retirement age but also later in old age (Dahlberg et al., 2007; Ophir, 2022). However, as countries moved to raise retirement age, an increasing share of women in their “encore years” are postponing retirement and remaining in full-time employment. This may reduce their availability to provide care outside their household (Rodrigues & Ilinca, 2021). In addition, current cohorts of women approaching retirement age have experienced a combination of delayed childbearing and increased longevity of their older relatives often spent in poor health, resulting in greater shared life years with older parents, as well as adult children (Moen, 2016; Patterson & Margolis, 2019). The burdens of dual caregiving roles could put pressure on older women of working age to provide upward or downward intergenerational care, even when they live outside multigenerational households. Declining marriage rates and increasing divorces across these age groups may have a different impact on caregivers. Among older working-age people (ONS, 2017), increasing divorces may unburden women from the obligation to provide care for their in-laws. Shifts in marriage and divorce patterns observed after retirement, together with a higher share of childless older people, could render horizontal care provided to friends or neighbors more relevant—a type of non-cohabiting care that is also more likely to be provided by women (Patterson & Margolis, 2019).

For older age groups, there is a closing or even reversal of the gender care gap (Ophir, 2022; Ophir & Polos, 2022), particularly in spousal care (Glauber, 2017; Hirst, 2001; Patterson & Margolis, 2019)—a pattern that seems to have held over time (Arber & Ginn, 1992, 1995; Dahlberg et al., 2007). Among men, retirement produces a reallocation of time away from full-time employment and into spousal care (Glauber, 2017), resulting in greater care intensity (Dahlberg et al., 2007). Men may be more likely to provide spousal care at later ages because of the “gender health paradox,” whereby women outlive men, but live the extra years in poor health, thus needing care. However, there are signs that the gender gap in spousal care may also be shifting along the different stages of old age. For example, as the gender gaps in seniority and earnings diminish, the opportunity costs of providing spousal care in the years leading up to retirement may increase for women (Glauber, 2017). The above-mentioned rising trend in divorces has also been observed in older age groups and this could further contribute to reducing the available pool of spousal carers. This latter effect may be counterbalanced, however, by greater cohabitation in old age that has been witnessed in the past decades (Brown & Wright, 2017). Similar contradictory effects are grounded in demographics. On the one hand, the gender gap in life expectancy has steadily diminished (HMD, 2015), potentially resulting in higher availability of male spousal carers in older age groups. On the other hand, there is evidence that this may be accompanied by declining functional ability in old age among surviving men (Fors et al., 2022), which could increase the demand for caregiving from female spouses.

Another key factor in existing gender inequalities in informal caregiving in Europe is institutions, more specifically, the welfare state. These affect the gender care gap through the generosity and design of their social benefits, which attribute different care responsibilities to families and indirectly to women (Albertini et al., 2007). Although typologies of care regimes abound with different constellations of countries, there is some consistency in the North–South dichotomy of welfare state generosity and caregiving arrangements in Europe (Bolin et al., 2008; Brandt et al., 2009; Sarasa & Billingsley, 2008). In Nordic European countries, care policies prioritize gender equality and reduction of reliance on the family for meeting the care needs of adults. This is done through the provision of generous public care services. As a result, caregiving is more equally shared between women and men and is less intensive. In Southern Europe, care services for adults in need of care are limited and the family, particularly women, are expected to act as default caregivers (Bettio & Plantenga, 2004). In these countries, caregiving is concentrated on women and it is generally of an intense nature. The Continental cluster (including Austria, Germany, and France, for example), sits somewhere in between Nordic and Southern care regimes in terms of gender equality in informal care (Bettio & Plantenga, 2004; Schmid al., 2012). Families are supported in their caregiving role through cash-for-care benefits. As these benefits more often influence the caregiving behavior of women but not of men (Haberkern et al., 2015), caregiving is concentrated among women and is generally of an intense nature, albeit less than in Southern Europe. This picture is not static. In the past decades, the availability and affordability of care services have changed across care regimes. Countries in Continental Europe have expanded the provision of formal care for older people, whereas in Northern Europe, generosity of care services has been concentrated on those with higher care requirements (Ranci & Pavolini, 2015). The expansion of services in countries that have traditionally relied on the family to provide care, such as those in the Continental cluster, is more likely to have freed women from caring duties. Welfare retrenchment in Nordic countries seems to have been compensated for by an increase in caregiving (Ranci & Pavolini, 2015), although the effect on the gender care gap in such egalitarian countries is difficult to anticipate.

To our knowledge, only one previous study on England attempted to analyze changes in informal caregiving to adults over time from a gender perspective (Hirst, 2001). The findings showed a closing gender care gap in both care provided outside and inside the household. Although the former arose from a sustained drop in women’s involvement in that type of care, the latter was mostly driven by increased uptake of spousal care by men. Hirst’s study, however, was based on repeated cross-sectional data and thus unable to account for changes in caregiving trajectories among different age groups of carers. Dynamic cohort analysis would allow for a better exploration of changes across groups marked by different contexts and trajectories along their life course.

Our study answers three main research questions. The first is whether there has been a change in the gender gap in informal caregiving across cohorts. A related second research question refers to whether this is due to changes in informal caregiving by women and/or men. Based on the variation of care policies and how they have evolved across European countries, our third research question is whether the gender gap in informal caregiving has narrowed or widened across different care regimes.

## Data and Methods

### Sample

Data were extracted from panel waves 1, 2, 4, 5, and 6 of the Survey of Health, Ageing and Retirement in Europe (SHARE), a cross-national longitudinal survey that includes information on support and informal care in the community. Data include 11 European countries that together represent 63% of the population of the European Union and Switzerland in 2015 and cover the time span between 2004 and 2015. We included countries that participated in at least three waves, except for Poland and the Czech Republic, due to limited sample sizes. We maintained in the analytical sample only those individuals who entered the SHARE sample in the first or second wave (2004 and 2007, respectively), were aged 50 or over at the time of the first interview, who participated in at least three panel waves overall, and who provided valid responses for all included variables.

The individuals who fulfilled the inclusion criteria were grouped into six birth cohorts, spanning 5-year intervals for those born between 1930 and 1954. To ensure sufficient sample size, the oldest birth cohort included all individuals born before 1929. For regional analyses, we further grouped individual observations into three country clusters (commonly referred to as care regimes) that have been previously found to reflect similarities within those in the same group (Albertini & Pavolini, 2017; Carrieri et al., 2017):

1. Continental (Austria, Germany, France, Switzerland, Belgium, and the Netherlands)
2. Southern (Spain, Italy, and Greece)
3. Nordic (Sweden and Denmark)

The final analytic sample included 78,607 observations, 55.5% of which belong to women, from 25,480 individuals.

### Measures

Informal caregiving was initially defined as a binary variable, with positive responses for those who reported (a) having helped regularly with personal care, such as washing, getting out of bed, or dressing another person living in the same household, and/or (b) having given personal care or practical household help to a family member living outside their household, a friend, or neighbor in the last 12 months. In our analysis, we also differentiated between provision of informal care inside and outside the household, both defined as binary variables, taking the definitions outlined under points (a) and (b), respectively. The former is largely characterized by spousal care, comprising 68% of this care in our sample. As a large body of literature shows an inverse relationship between intensity and probability to provide care for adults across Europe (Albertini et al., 2007; Schmid et al., 2012), we also defined a variable for intense caregiving, operationalized as providing daily care outside the household versus those providing it less often or not at all. Care inside the household is assumed to be daily; therefore, we restrict our analysis of intensity to care outside of the household. In our definition of caregiving, we account for upward (e.g., to parents), horizontal (e.g., to friends, siblings, or spouses), and downward (e.g., to adult children) care. Given the focus on caregiving to adults with care needs, we excluded childcare provided to grandchildren (i.e., grandparenting), which in SHARE is captured by a separate question.

Additional covariates were included based on the literature on determinants of informal care and reflected possible changes in the past decades that could have affected the gender care gap. These included self-reported health (categorical through a five-element Likert scale increasing in value: excellent, very good, good, fair, and poor), number of chronic conditions, respondent’s highest educational achievement (categorical variable: primary, secondary, tertiary), current employment status (binary variable operationalized as having any type of employment as opposed to not being employed), and the presence of a partner living in the same household as the respondent (binary variable operationalized as living with partner or without one). “Time” measured as the elapsed calendar year between the first wave and the interview date, gender (binary variable: men and women) and birth cohort were also included. We also include the number of limitations with activities of daily living as a proxy for “need” of the cared-for person, but as this is asked only for the interviewed spouse, we show this as a sensitivity analysis for a subsample of spousal care (Supplementary Material 1).

### Analytic Strategy

Adapting Marshall and colleagues’ (2015) model for frailty trajectories across cohorts to informal care, we first constructed mixed-effects logistic regression models to estimate the probability of providing informal care. The model includes a random effect in Level 1 (i.e., for the individual’s caregiving trajectories) for each survey wave to account for individual heterogeneity and trajectories in providing informal care. All the other independent variables were entered into the model as fixed effects in Level 2 (i.e., trajectories across individuals). This methodological approach affords us two advantages: (1) to account for the unbalanced nature of our panel as not all respondents have full information for all waves and (2) to account for individual-level variability in the decision to provide care. Failing to consider the latter point would result in biased standard errors. All models are specified to include a main effect for gender, cohort, and time, as well as interaction terms between them, which allow for different slope estimates across cohort, time, and gender. We further include a quadratic term for time effects to capture nonlinearity in individual slopes.

We estimate both unadjusted models which capture the effects of time, cohort, and gender, and adjusted models which further account for the control variables described previously. The results are presented as average marginal effects (AMEs) for ease of interpretation across models and are calculated as the difference in estimated probabilities by gender over each cohort. To disentangle age and cohort effects, we additionally present graphs of the trajectories of caregiving care across gender and cohort using predicted probabilities from the adjusted models described previously (Marshall et al., 2015; Rogers et al., 2017), in order to compare cohort differences across overlapping ages (underlying models and predicted probabilities available in Supplementary Material 2). In the graphs, AMEs are presented for each age; however, the individual trajectories observed for each individual are based on the data intervals of SHARE (collected between 2- and 4-year intervals). Throughout the analysis, we used calibrated cross-sectional individual weights, calculated for the entire survey sample at the baseline wave in which each individual joined the sample, to account for attrition. All analyses were performed using STATA version 15.

## Results

Table 1 presents selected sample characteristics across Waves 1–6 (2004–2015) for each of the cohorts considered across the age ranges in which they were observed in the sample. Within each cohort, the prevalence of caregiving decreases with age for women and men in the sample. Women in the three later-born cohorts (1950–1954, 1945–1949, and 1940–1949) have a higher prevalence than men of providing informal care either inside and outside the household across nearly all periods or waves, whereas in the three earlier-born cohorts, the prevalence of informal caregiving among men is progressively higher in comparison to that of women. However, cohorts are observed at different ages across Waves 1 and 6. Observed differences may thus reflect genuine cohort differences or/and simply the different stages in the life course in which cohorts are observed (i.e., they might simply be the effect of age). Comparisons across cohorts are more meaningful for the age ranges in which they overlap: compare, for example, caregiving probabilities for women and men observed in Wave 4 for those born in 1950–1954 with those observed in Wave 2 for those born in 1945–1949.

**Table 1.** Descriptive Statistics of the Analytical Sample for 11 European Countries, 2004–2015

Cohort		Survey wave, year				
		Wave 1	Wave 2	Wave 4	Wave 5	Wave 6
		2004	2007	2011	2013	2015
1950–1954	Men (*n*)	1,741	1,934	954	938	1,297
	Women (*n*)	2,092	2,371	1,220	1,216	1,629
	Mean age (years)	52.3	54.9	59.1	61.1	63.1
	Gives care (% of males)	45.26	41.21	40.04	40.30	37.24
	Gives care (% of females)	47.75	49.18	45.98	46.13	40.52
1945–1949	Men (*n*)	1,804	2,180	1,006	996	1,395
	Women (*n*)	2,213	2,498	1,320	1,302	1,633
	Mean age (years)	57.1	59.7	64.0	66.0	67.9
	Gives care (% of males)	42.07	41.15	38.57	40.86	34.84
	Gives care (% of females)	46.72	42.79	41.74	42.78	36.37
1940–1944	Men (*n*)	1,628	1,884	938	846	1,187
	Women (*n*)	1,868	2,201	1,137	1,088	1,403
	Mean age (years)	62.1	64.7	68.9	71.0	72.9
	Gives care (% of males)	40.72	37.26	33.26	35.22	31.00
	Gives care (% of females)	40.20	40.80	41.07	38.14	27.87
1935–1939	Men (*n*)	1,486	1,724	831	729	1,014
	Women (*n*)	1,640	1,832	966	919	1,122
	Mean age (years)	67.1	69.6	73.9	75.9	77.9
	Gives care (% of males)	36.74	36.66	30.20	29.49	26.33
	Gives care (% of females)	36.16	32.91	33.95	33.30	22.82
1930–1934	Men (*n*)	1,148	1,346	618	552	682
	Women (*n*)	1,321	1,461	808	746	832
	Mean age (years)	72.1	74.6	78.9	80.9	82.9
	Gives care (% of males)	30.49	27.93	27.51	25.18	20.09
	Gives care (% of females)	27.33	25.26	26.98	25.74	17.31
1900–1929	Men (*n*)	1,370	1,522	574	427	400
	Women (*n*)	1,998	2,159	1,021	765	675
	Mean age (years)	80.2	82.6	86.2	87.7	89.3
	Gives care (% of males)	22.99	23.72	24.39	20.84	18.25
	Gives care (% of females)	19.82	17.09	18.81	15.56	11.70

*Note*: Non-weighted results.

Table 2 presents the AMEs for gender differences in informal caregiving (i.e., the gender care gap) across cohorts for overlapping age ranges, for the unadjusted and adjusted models. Among younger age groups, where women are more likely to provide informal care than men, the gender care gap widens for later-born cohorts (those born in or after 1945, for the unadjusted model, and in or after 1940, for the adjusted model). For older age ranges, the gender care gap is reversed, but this difference loses significance for later-born cohorts.

**Table 2.** Average Marginal Effects for Women Versus Men for Providing Informal Care Across Cohorts for Overlapping Ages, Pooled Sample (11 European Countries), 2004–2015

	AME for women					
	Cohort 1950–1954	Cohort 1945–1949	Cohort 1940–1944	Cohort 1935–1939	Cohort 1930–1934	Cohort 1900–1929
Age group	Unadjusted model					
57–63	0.059***	0.055***	—	—	—	—
62–68	—	0.053***	0.024	—	—	—
67–73	—	—	0.024	−0.008	—	—
72–78	—	—	—	−0.008	−0.012	—
80–83	—	—	—	—	−0.012	−0.056***
Age group	Adjusted model					
57–63	0.067***	0.064***	—	—	—	—
62–68	—	0.063***	0.040**	—	—	—
67–73	—	—	0.040**	0.018	—	—
72–78	—	—	—	0.017	0.009	—
80–83	—	—	—	—	0.009	−0.033**
Number of observations (number of groups)		78,607 (25,480)				

*Notes*: Unadjusted model includes only gender, cohort, and time, as well as interactions between these variables. Adjusted models include also as covariates partner living in the household, self-rated health, education, employment, and number of chronic conditions. Estimated using a mixed-effects logistic regression model. Log likelihood for unadjusted and adjusted models are, respectively, −179,300,000 and −177,700,000. AME = average marginal effect.

*p < .05. **p < .01. ***p < .001.

**Figure 1.**
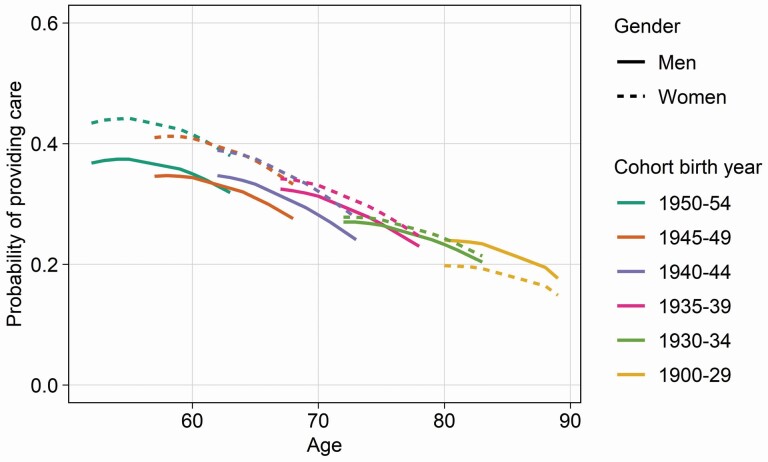
Estimated probabilities of giving care, by gender in 11 European countries, 2004–2015 (from adjusted mixed-effects logistic regression models). *Notes*: Models adjusted for partner living in the household, self-rated health, education, employment, and number of chronic conditions. Predicted probabilities available in Supplementary Material 2. Weighted results.

Table 2 shows a widening of the gender care gap for later-born cohorts for some age ranges, but it still does not allow for a clear differentiation of the underlying caregiving trajectories. Figure 1 provides a graphic representation of the informal caregiving trajectories for each cohort based on the adjusted model, for women and men separately, over the 11-year period in which they are observed in our sample and across different age ranges. This graphic representation allows for a concrete differentiation between cohort and age effects, by displaying predicted probabilities to provide informal care across overlapping age ranges for different cohorts. For women, we observe that caregiving declines across age groups (i.e., an age effect) with only limited differences between cohorts for overlapping age ranges, that is, the probability to provide informal care does not change significantly across cohorts. The exception is the women born between 1930 and 1934, for whom the probability to provide care increased in comparison to  the previous cohort for overlapping age ranges. For men, the  individual prevalence of informal caregiving also decreases with age (albeit in a less steep manner than for women), but Figure 1 also shows that these decreases are observed progressively lower in later-born cohorts due to a cohort effect. For overlapping ages, the distance between caregiving trajectories of later-born cohorts of women and men is larger than for earlier-born cohorts, denoting a widening of the gender care gap.

**Figure 2.**
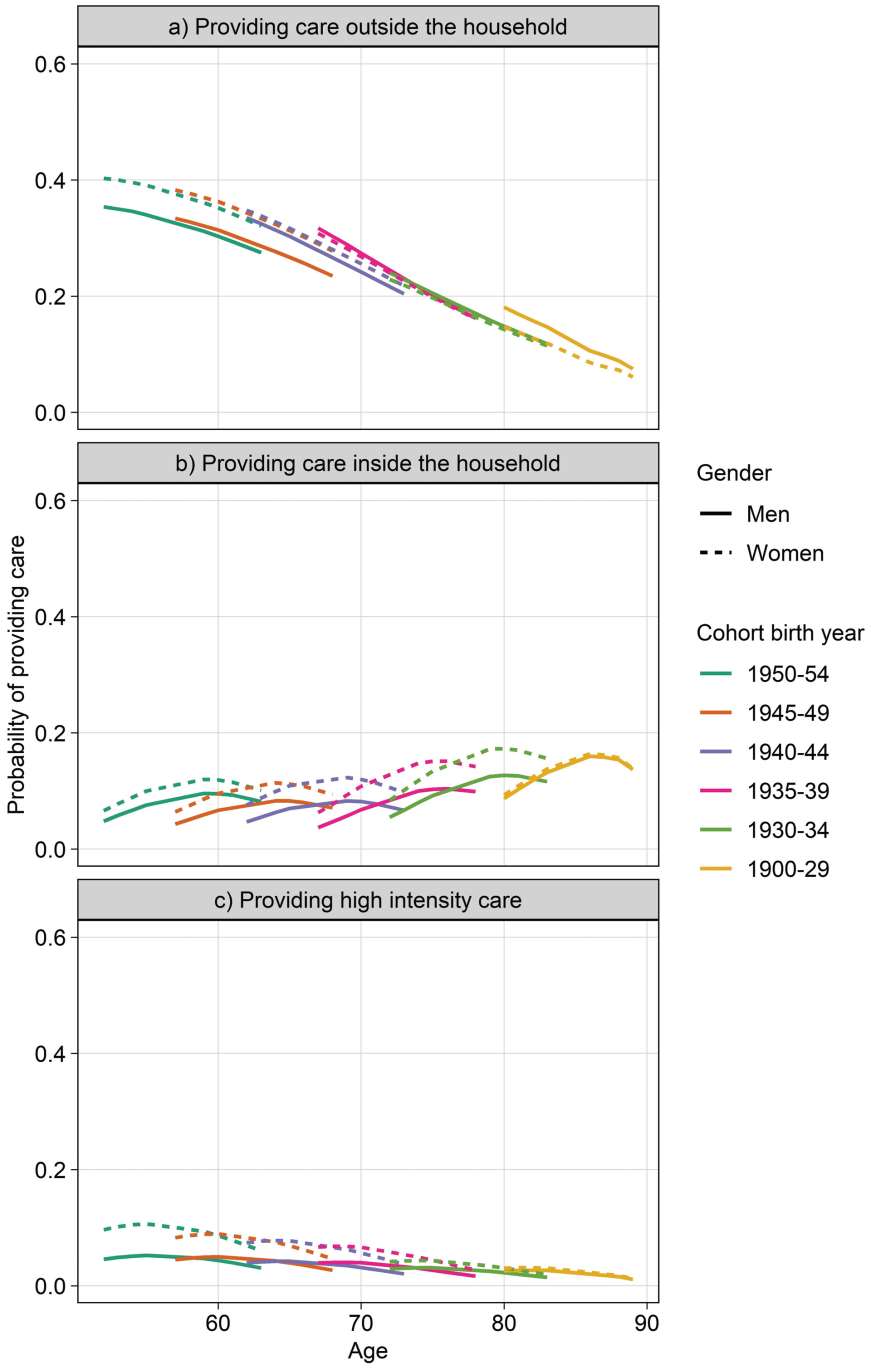
(A–C) Estimated probabilities of giving care outside and inside the household and for giving intense care outside the household, by gender in 11 European countries, 2004–2015 (from adjusted mixed-effects logistic regression models). *Notes*: Models adjusted for partner living in the household, self-rated health, education, employment, and number of chronic conditions. Predicted probabilities available in Supplementary Material 2. Weighted results.

In the age ranges for which the cohorts born in 1940 or after are included in our sample, informal care is likely to be intergenerational care provided outside the household, whereas in earlier-born cohorts, spousal care is much more prevalent. Figure 2A and B present the informal caregiving trajectories by cohort, disaggregated by the type of informal care provided for the adjusted model. For informal care provided outside the household (Figure 2A), we observe again a permanence of its prevalence across cohorts for women (i.e., we observe only age effects). Conversely, there is a pronounced decrease in this same prevalence for men for the age groups where cohorts overlap (i.e., the distance between cohort lines across overlapping ages within men is wider), particularly for the cohorts born between 1940 and 1949. There is, therefore, a widening gender care gap in this type of care for later-born cohorts. For care inside the household (Figure 2B), its prevalence increases among later-born cohorts for both men and women, without any clear change in the gender care gap. Given changes in the morbidity of older persons across the time period, we carry out a sensitivity analysis controlling for the needs of the cared-for person in a subsample of spousal care (Supplementary Material 1). Accounting for spousal health affects the probability to provide spousal care across age groups (i.e., age effect) predominantly for women, leading to a narrower gender gap, particularly for those between the ages of 70 and 80. Cohort effects remain even after controlling for needs. Figure 2C presents informal caregiving trajectories by cohort for intense caregiving. The gender gap in intense caregiving disappears entirely around the age of 80, explained primarily by the reduction in intense care by women as they age, as the prevalence of intense care among men is mostly flat across age groups. The prevalence of intense caregiving decreases among later-born cohorts for similar age groups, and although this is more visible for women (especially below the age of 70), this cohort effect is of limited magnitude.

**Figure 3.**
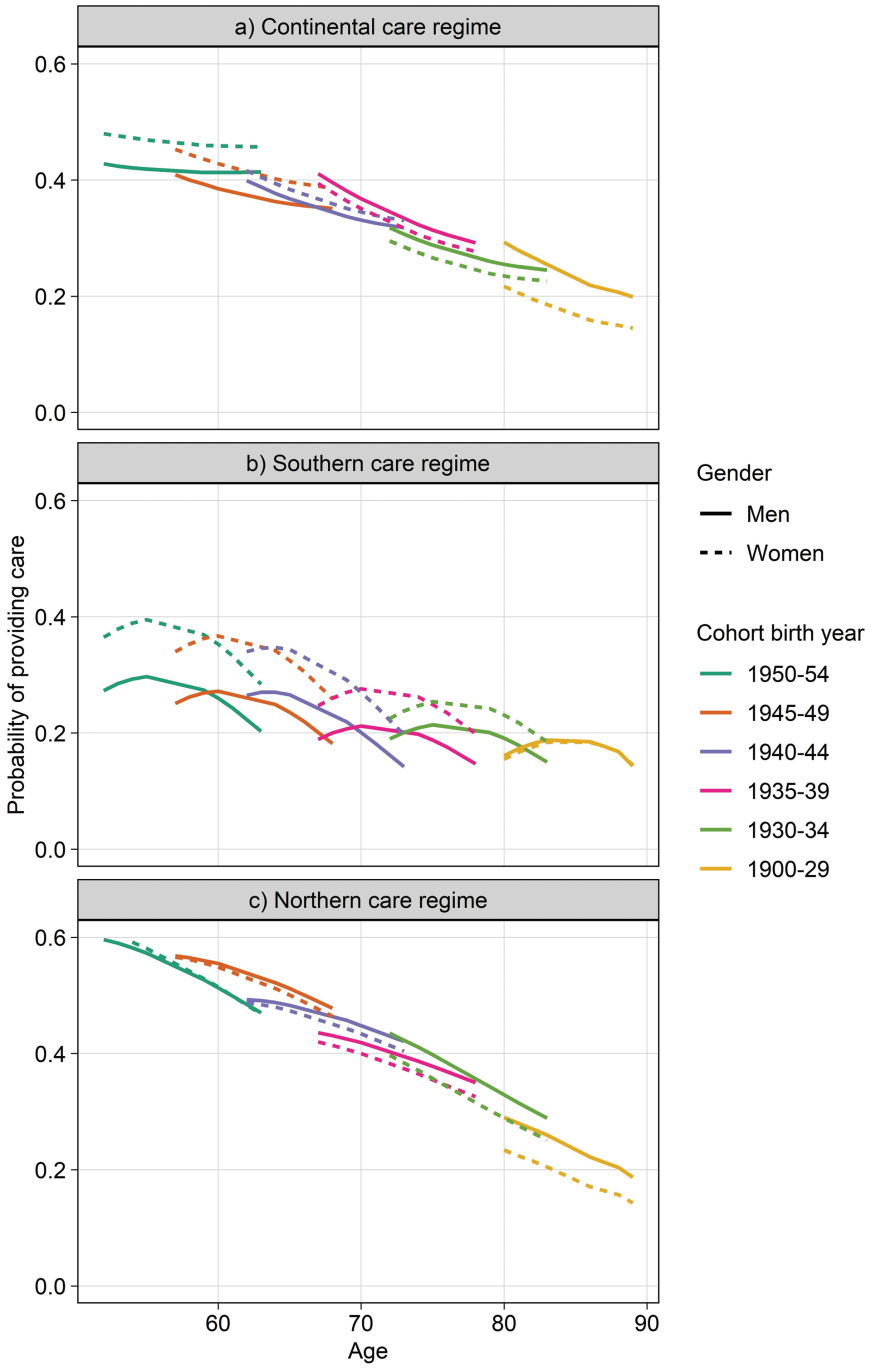
(A–C) Estimated probabilities of giving care, by gender and care regime, 2004–2015 (adjusted mixed-effects logistic regression models). *Notes*: Models adjust for partner living in the household, self-rated health, education, employment, and number of chronic conditions. Continental includes Austria, France, Germany, the Netherlands, Switzerland, and Belgium; Southern includes Spain, Italy, and Greece; Northern includes Denmark and Sweden. Predicted probabilities available in Supplementary Material 2. Weighted results.

Next, we evaluate possible variations across different care regimes by running the final adjusted model for the probability to provide any type of informal care for each of the three care regimes separately. Figure 3 shows pooled informal care trajectories for age and cohort by care regime. In the Continental cluster, for similar age ranges, women of later-born cohorts are more likely to provide informal care. For men, there is no clear cohort trend, resulting in a widening gender gap for later-born cohorts. In the Southern care cluster, there is a reduction in the probability to provide informal care among later-born cohorts where age overlaps across cohorts. This pattern is similar for both women and men. For the Northern cluster, there is no clear cohort trend for men, whereas for women, there appears to be an increase in the prevalence of informal caregiving for later-born cohorts, especially in older age groups where cohorts overlap and when caregiving is more likely to be spousal care. As men show a higher probability to provide informal care in those age ranges (age effect), the result is a narrowing of the gender gap for more recent-born cohorts.

## Discussion

This study makes a novel contribution to the literature on informal caregiving and gender inequalities by analyzing gendered cohort trajectories of informal caregiving to adults with care needs along the later stages of the life course across Europe. Regarding the first research question—whether there has been a change in the gender gap in informal caregiving across cohorts—we find a widening gender care gap for later-born cohorts at earlier age ranges. Trajectories of informal caregiving by gender—our second research question—uncover different cohort developments between women and men after accounting for several covariates. For care outside the household, we observe only an age effect among women, whereas there is an increase in the prevalence of care provided to co-residing older people for later-born cohorts of women where age ranges overlap (i.e., a cohort effect). For men, later-born cohorts show a lower probability of providing informal care across overlapping age ranges, but this masks opposite developments in care inside and outside the household. The likelihood of providing care inside the home has increased for later-born cohorts while decreasing outside the household, the latter especially for age groups below or around the age of 70. The gender care gap, therefore, seems to be widening among later-born cohorts because of men’s lower probability of providing informal care over time, particularly outside the household. We find similar age effects to other studies (Glauber, 2017; Ophir, 2022; Patterson & Margolis, 2019), namely that women are more likely than men to provide informal care in the encore years of adulthood, with the pattern being reversed for the oldest age groups. This reversal of the gender care gap for care provided outside the household used to happen much earlier and has been delayed for later-born cohorts due to the cohort effects we show for men. The decreasing likelihood of caregiving outside the household for later-born cohorts of men could be linked to increases in statutory retirement age that have led to the postponement of the age at which men shift their time to unpaid caregiving (Rodrigues & Ilinca, 2021). The occurrence of caregiving to someone outside the household has apparently remained the same among women, even when faced with similar increases in the statutory retirement age. This could be caused by women having to respond to increased demand for multigenerational care (i.e., combination of upward, horizontal, and/or downward care), as periods of shared lives broaden because of delayed fertility and increasing life expectancy of older relatives (Patterson & Margolis, 2019). An analysis on changes in care intensity for care outside the household suggests that there was, nonetheless, an adjustment as intensive caregiving is less prevalent among later-born cohorts of women, especially below the age of 70. This result is consistent with studies analyzing the effect of pension reforms on female supply of caregiving (Bergeot & Fontaine, 2020; Rodrigues & Ilinca, 2021). Adjustments in terms of intensity also appear contradictory: higher intensity care outside the household decreased, but seems to have been compensated for by care provided inside the household (which by definition in SHARE could be considered high intensity). The rising prevalence of caregiving inside the household for both men and women—which in our sample is mostly comprised of spousal care for both sexes—seems to be driven primarily by demographic developments. With a narrowing of the sex/gender differential in life expectancy, the share of older people living as cohabiting couples has steadily increased relative to those living alone, even after accounting for increasing gray divorces (ONS, 2017). This has rendered spousal care, which makes up the majority of care inside the household, more available than before. Our findings show that besides increased cohabitation in older age, morbidity may also play a role. Prevalence of spousal care in later-born cohorts is significantly reduced and the gender care gap essentially halved after accounting for the health condition of the cared-for spouse, especially among later age groups. This suggests that increased longevity with poorer health among men (Fors et al., 2022) is not only increasing the availability of male carers in later-born cohorts, but also increasing the demand for spousal care at later stages of the life course.

Our third research question was whether the gender gap in informal caregiving has narrowed or widened across different care regimes. Findings confirm the existence of dissimilar trends across different clusters of European countries. After accounting for socioeconomic conditions, health, and living arrangements, women in Continental Europe are more likely to provide informal care in later-born cohorts than in earlier ones where age ranges overlap, resulting in a widening gender care gap. Among Southern European countries, later-born cohorts are less likely to provide informal care, but this cohort effect is similar for women and men across age groups, with no visible effect on the gender care gap. The Northern cluster has the least gender differences in the prevalence of caregiving, with women of later-born cohorts increasingly more likely to provide care in older age groups where spousal care is predominant and men are more likely to provide care, resulting in a narrowing of the gender care gap. The only care regime that shows increasing caregiving among later-born cohorts in overlapping age groups up till retirement is the Continental cluster. As provision of care services and statutory retirement age both increased during this time period for most countries in the Continental and Southern clusters, the apparent contradictory developments in caregiving are surprising. These effects were observed through a period of societal and institutional upheaval due to the Great Recession, which may have families’ ability to provide support, including caregiving (Tur-Sinai et al., 2020). This is, nonetheless, an area that merits further research.

Our findings have differentiated policy implications across several stages of the life course. The stability or even increase in the prevalence of female caregiving across cohorts (in the case of care outside the household) in the years leading up to statutory retirement seems to contradict the until now largely untested proposition that increased education and employment would reduce the number of female carers. On the one hand, policies to increase female labor market participation, such as increased pension age, have apparently not significantly reduced women’s caregiving around retirement age (Bergeot & Fontaine, 2020). During this same period, however, some countries rolled back care services (Ranci & Pavolini, 2015), which may have left women having to cope with multiple roles at work and at home (Marks, 1996). On the other hand, given that these findings were mostly confined to women—men in later-born cohorts are much less likely to provide care outside the household till retirement age—this raises the issue of whether policies aimed at full-time employment or prolongation of (paid) working lives sufficiently account for the “second shift” still carried out by women through unpaid care in their encore years of adulthood (Ophir, 2022).

The increasing relevance of care inside the household after retirement, which in our sample, closely follows spousal care, however, entails different challenges in terms of care support. First of all, we show that this may be associated with poorer health of the cared-for person, which could entail a greater burden of care. Finally, the permanence of the gender care gap throughout these later life stages questions the effectiveness of policies aimed at achieving greater gender equality in care. Following the example of the Nordic countries, which achieved a modicum of gender equality in caregiving in later-born cohorts, greater service availability and an overall gender equality-friendly societal context (e.g., lower gender gaps in earnings and employment) are still needed.

This study relies on a large, harmonized data set with several points of data collection, providing sufficient power to analyze gender differences even at higher age groups, while accounting for within cohort heterogeneity. One remaining concern, however, is SHARE’s definition for informal caregiving inside the household. This is limited to personal care, which could underestimate overall prevalence, although what this means for the gender care gap is not straightforward. By excluding help with household tasks as well as emotional care, both predominantly carried out by women, the definition employed by SHARE would risk underestimating women’s care provision (Patterson & Margolis, 2019; Rutherford & Bu, 2018). Response rates for the baseline ranged between 36.4% and 92.7%. Nonresponse may vary across health and income, which in turn correlate with caregiving. The longitudinal nature of this study and sample exclusion criteria may further exacerbate nonresponse bias. We used the calibrated cross-sectional weights provided by SHARE to account for selective nonresponse and to minimize their impact on estimates. Furthermore, nonresponses are not systematically concentrated among particular regional clusters (Supplementary Material 3), minimizing their impact on the care regime analysis. Finally, as in other cohort studies, there is only a partial overlap across age ranges (cf. Fors et al., 2022; Marshall et al., 2015). In the future, a more detailed analysis of cohort effects could be accomplished when further waves of the data become available.

This study addresses Moen and DePasquale’s (2017, p. 46) call for research that increases our knowledge of “the multiple ways in which family care plays out over time,” by analyzing changes across the later stages of the life course, as well as changes across cohorts. It reports on cohorts up to the oldest baby boomers, whose formative years were characterized by expansion of educational and employment opportunities for women and shifts in fertility regimes. Once data on more recent-born cohorts become available, it will become apparent whether the gender roles that we have found among the cohorts included in this study have become permanent.

## Supplementary Material

gbad011_suppl_Supplementary_Material_S1

gbad011_suppl_Supplementary_Material_S2

gbad011_suppl_Supplementary_Material_S3
