# Supplementary material for: Cohort Trajectories by Age and Gender for Informal Caregiving in Europe Adjusted for Sociodemographic Changes, 2004 and 2015
Source: J Gerontol B Psychol Sci Soc Sci. 2023 Jan 23;78(8):1412–22. doi: 10.1093/geronb/gbad011 (PMC10394995; doi:10.1093/geronb/gbad011)
Supplement: gbad011_suppl_Supplementary_Material_S1 [file gbad011_suppl_supplementary_material_s1.docx]

**Supplementary Figure 1**: Estimated probabilities of providing informal care to a spouse according to spousal health, 2004-2015


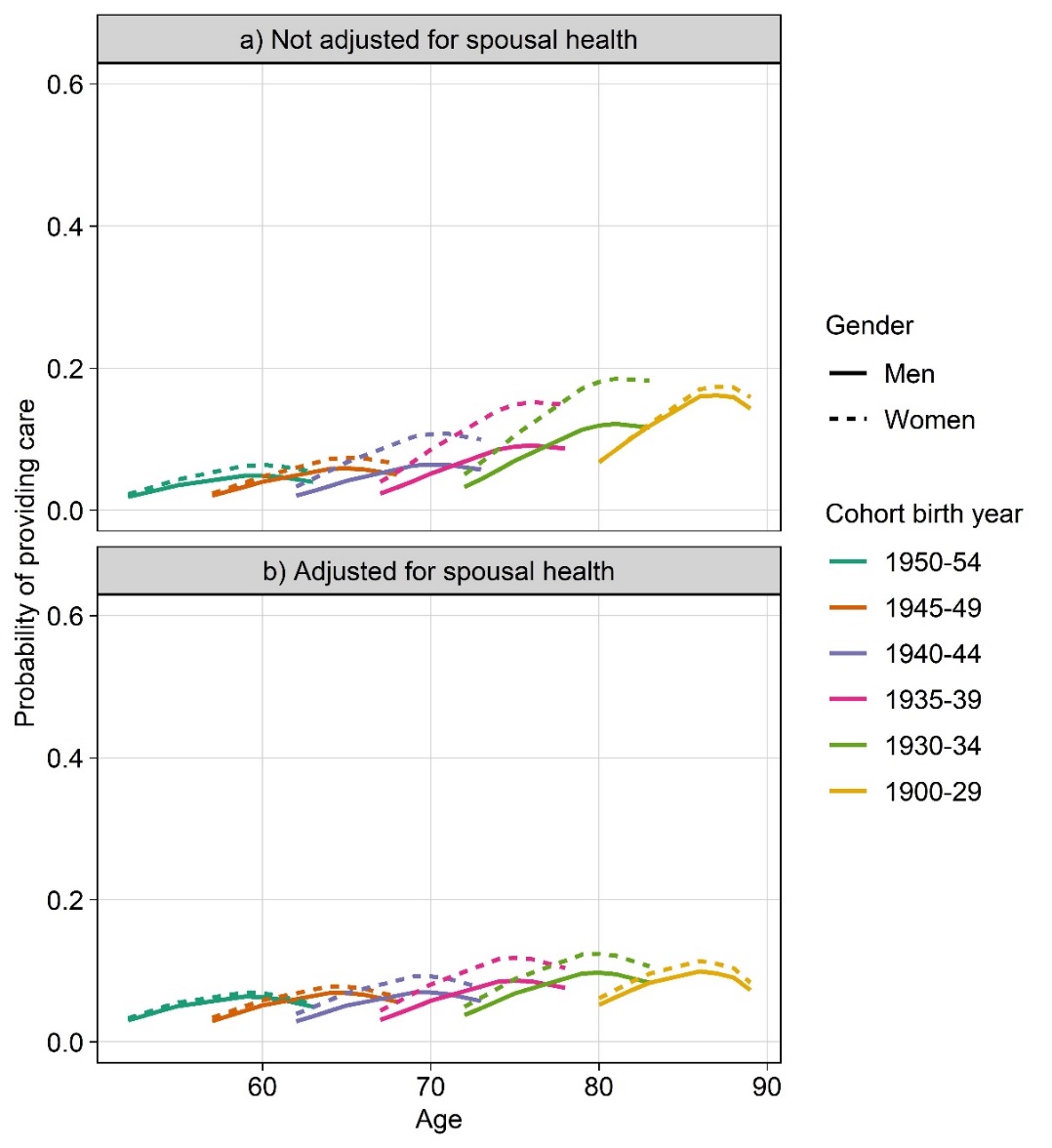


Note: Spousal health (graph b) defined as a continuous variable of the number of self-reported ADL limitations models. Models in addition control for partner living in the household, self-rated health, education, employment and no. of chronic conditions of carer. Model used for estimation is a random effects logistic regression, not strictly comparable with the models in Figures 1-3 in the manuscript as it does not allow for temporal variations in the random slopes. Weighted results.
