# Supplementary material for: Cohort Trajectories by Age and Gender for Informal Caregiving in Europe Adjusted for Sociodemographic Changes, 2004 and 2015
Source: J Gerontol B Psychol Sci Soc Sci. 2023 Jan 23;78(8):1412–22. doi: 10.1093/geronb/gbad011 (PMC10394995; doi:10.1093/geronb/gbad011)
Supplement: gbad011_suppl_Supplementary_Material_S2 [file gbad011_suppl_supplementary_material_s2.docx]

**Supplementary Table 1:** Mixed effects logistic regression for informal caregiving (all types) (underlying model for Figure 1). Pooled sample (LCI, lower confidence interval; UCI, upper confidence interval)

|  | Odds-Ratio | P-value | LCI | UCI |
| --- | --- | --- | --- | --- |
| **Fixed Effects** |  |  |  |  |
| Sex (ref=male) | 1.412 | 0.000 | 1.189 | 1.676 |
| Cohort (ref= 1950-54) |  |  |  |  |
| 1945-49 | 0.889 | 0.219 | 0.736 | 1.073 |
| 1940-44 | 0.894 | 0.257 | 0.736 | 1.085 |
| 1935-39 | 0.789 | 0.024 | 0.642 | 0.969 |
| 1930-34 | 0.572 | 0.000 | 0.457 | 0.716 |
| 1900-29 | 0.472 | 0.000 | 0.374 | 0.595 |
| Time | 1.026 | 0.097 | 0.995 | 1.057 |
| Cohort*time (ref= 1950-54) | |  |  |  |
| 1945-49 | 0.985 | 0.159 | 0.964 | 1.006 |
| 1940-44 | 0.962 | 0.000 | 0.941 | 0.983 |
| 1935-39 | 0.966 | 0.003 | 0.943 | 0.988 |
| 1930-34 | 0.976 | 0.067 | 0.951 | 1.002 |
| 1900-29 | 0.972 | 0.058 | 0.944 | 1.001 |
| Gender*time | 1.003 | 0.678 | 0.989 | 1.017 |
| Cohort*gender (ref= 1950-54) | |  |  |  |
| 1945-49 | 0.996 | 0.972 | 0.809 | 1.227 |
| 1940-44 | 0.885 | 0.259 | 0.715 | 1.094 |
| 1935-39 | 0.778 | 0.026 | 0.624 | 0.971 |
| 1930-34 | 0.744 | 0.019 | 0.582 | 0.952 |
| 1900-29 | 0.525 | 0.000 | 0.408 | 0.676 |
| Time*time | 0.994 | 0.000 | 0.992 | 0.997 |
| Education (ref=Primary) |  |  |  |  |
| Secondary | 1.624 | 0.000 | 1.505 | 1.754 |
| Tertiary | 2.173 | 0.000 | 1.967 | 2.401 |
| Self-rated health (ref=Excellent) | |  |  |  |
| Very good | 0.866 | 0.016 | 0.771 | 0.974 |
| Good | 0.796 | 0.000 | 0.712 | 0.889 |
| Fair | 0.702 | 0.000 | 0.620 | 0.795 |
| Poor | 0.551 | 0.000 | 0.471 | 0.644 |
| Partner in household (ref=no) | 0.768 | 0.000 | 0.710 | 0.831 |
| Employed (ref=no) | 0.931 | 0.142 | 0.846 | 1.024 |
| Chronic condition | 1.074 | 0.000 | 1.050 | 1.099 |
| Intercept | 0.457 | 0.000 | 0.375 | 0.559 |
| **Random Effects** |  |  |  |  |
| Wave | 0.009 |  | 0.007 | 0.012 |
| Intercept | 1.132 |  | 1.023 | 1.252 |
| No. of observations: 78,607 |  | No. of individuals: 25,480 | | |

**Supplementary Table 2a:** Mixed effects logistic regression for care outside of the household (underlying model for Figure 2a). Pooled sample (LCI, lower confidence interval; UCI, upper confidence interval)

|  | Odds-Ratio | P-value | LCI | UCI |
| --- | --- | --- | --- | --- |
| **Fixed Effects** |  |  |  |  |
| Sex (ref=male) | 1.318 | 0.003 | 1.101 | 1.579 |
| Cohort (ref= 1950-54) |  |  |  |  |
| 1945-49 | 0.896 | 0.277 | 0.736 | 1.092 |
| 1940-44 | 0.898 | 0.299 | 0.732 | 1.100 |
| 1935-39 | 0.808 | 0.056 | 0.649 | 1.005 |
| 1930-34 | 0.497 | 0.000 | 0.389 | 0.634 |
| 1900-29 | 0.318 | 0.000 | 0.244 | 0.415 |
| Time | 0.983 | 0.299 | 0.952 | 1.015 |
| Cohort*time (ref= 1950-54) | |  |  |  |
| 1945-49 | 0.984 | 0.149 | 0.963 | 1.006 |
| 1940-44 | 0.962 | 0.001 | 0.941 | 0.984 |
| 1935-39 | 0.939 | 0.000 | 0.916 | 0.962 |
| 1930-34 | 0.940 | 0.000 | 0.913 | 0.967 |
| 1900-29 | 0.923 | 0.000 | 0.892 | 0.956 |
| Gender*time | 1.003 | 0.683 | 0.988 | 1.019 |
| Cohort*gender (ref= 1950-54) | |  |  |  |
| 1945-49 | 0.999 | 0.995 | 0.805 | 1.241 |
| 1940-44 | 0.817 | 0.078 | 0.654 | 1.022 |
| 1935-39 | 0.721 | 0.006 | 0.570 | 0.913 |
| 1930-34 | 0.704 | 0.011 | 0.536 | 0.923 |
| 1900-29 | 0.563 | 0.000 | 0.418 | 0.758 |
| Time*time | 0.997 | 0.009 | 0.994 | 0.999 |
| Education (ref=primary) |  |  |  |  |
| Secondary | 2.116 | 0.000 | 1.943 | 2.303 |
| Tertiary | 2.965 | 0.000 | 2.661 | 3.302 |
| Self-rated health (ref=Excellent) | |  |  |  |
| Very good | 0.888 | 0.057 | 0.786 | 1.003 |
| Good | 0.785 | 0.000 | 0.700 | 0.881 |
| Fair | 0.599 | 0.000 | 0.526 | 0.683 |
| Poor | 0.313 | 0.000 | 0.262 | 0.374 |
| Partner in household (ref=no) | 1.155 | 0.001 | 1.063 | 1.255 |
| Employed (ref=no) | 0.919 | 0.093 | 0.833 | 1.014 |
| Chronic condition | 1.081 | 0.000 | 1.053 | 1.109 |
| Intercept | 0.325 | 0.000 | 0.264 | 0.401 |
| **Random Effects** |  |  |  |  |
| Wave | 0.007 |  | 0.005 | 0.011 |
| Intercept | 1.321 |  | 1.195 | 1.460 |
| No. of observations: 77,891 |  | No. of individuals: 25,477 | | |

**Supplementary Table 2b:** Mixed effects logistic regression for care inside of the household (underlying model for Figure 2b). Pooled sample (LCI, lower confidence interval; UCI, upper confidence interval)

|  | Odds-Ratio | P-value | LCI | UCI |
| --- | --- | --- | --- | --- |
| **Fixed Effects** |  |  |  |  |
| Sex (ref=male) | 1.469 | 0.022 | 1.057 | 2.043 |
| Cohort (ref= 1950-54) |  |  |  |  |
| 1945-49 | 0.861 | 0.442 | 0.587 | 1.261 |
| 1940-44 | 0.957 | 0.834 | 0.637 | 1.438 |
| 1935-39 | 0.721 | 0.122 | 0.476 | 1.092 |
| 1930-34 | 1.160 | 0.488 | 0.763 | 1.762 |
| 1900-29 | 2.141 | 0.000 | 1.425 | 3.217 |
| Time | 1.271 | 0.000 | 1.195 | 1.351 |
| Cohort*time (ref= 1950-54) | |  |  |  |
| 1945-49 | 0.994 | 0.761 | 0.954 | 1.035 |
| 1940-44 | 0.978 | 0.308 | 0.936 | 1.021 |
| 1935-39 | 1.057 | 0.012 | 1.012 | 1.105 |
| 1930-34 | 1.037 | 0.127 | 0.990 | 1.085 |
| 1900-29 | 1.005 | 0.841 | 0.957 | 1.056 |
| Gender*time | 0.994 | 0.650 | 0.969 | 1.020 |
| Cohort*gender (ref= 1950-54) | |  |  |  |
| 1945-49 | 1.116 | 0.585 | 0.753 | 1.655 |
| 1940-44 | 1.267 | 0.252 | 0.845 | 1.900 |
| 1935-39 | 1.307 | 0.199 | 0.868 | 1.967 |
| 1930-34 | 1.196 | 0.402 | 0.787 | 1.819 |
| 1900-29 | 0.741 | 0.157 | 0.489 | 1.123 |
| Time*time | 0.980 | 0.000 | 0.976 | 0.984 |
| Education (ref=primary) |  |  |  |  |
| Secondary | 0.814 | 0.002 | 0.714 | 0.927 |
| Tertiary | 0.664 | 0.000 | 0.552 | 0.798 |
| Self-rated health (ref=Excellent) | |  |  |  |
| Very good | 0.840 | 0.184 | 0.649 | 1.087 |
| Good | 1.066 | 0.614 | 0.833 | 1.364 |
| Fair | 1.450 | 0.006 | 1.113 | 1.890 |
| Poor | 1.980 | 0.000 | 1.464 | 2.677 |
| Partner in household (ref=no) | 0.955 | 0.635 | 0.788 | 1.156 |
| Employed (ref=no) | 0.695 | 0.000 | 0.573 | 0.842 |
| Chronic condition | 1.050 | 0.011 | 1.011 | 1.090 |
| Intercept | 0.019 | 0.000 | 0.012 | 0.030 |
| **Random Effects** |  |  |  |  |
| Wave | 0.014 |  | 0.008 | 0.023 |
| Intercept | 2.294 |  | 1.981 | 2.656 |
| No. of observations: 59,734 |  | No. of individuals: 21,069 | | |

**Supplementary Table 2c:** Mixed effects logistic regression for intensive care (underlying model for Figure 2c). Pooled sample (LCI, lower confidence interval; UCI, upper confidence interval)

|  | Odds-Ratio | P-value | LCI | UCI |
| --- | --- | --- | --- | --- |
| **Fixed Effects** |  |  |  |  |
| Sex (ref=male) | 2.556 | 0.113 | 2.047 | 3.192 |
| Cohort (ref= 1950-54) |  |  |  |  |
| 1945-49 | 0.983 | 0.132 | 0.759 | 1.273 |
| 1940-44 | 0.839 | 0.141 | 0.637 | 1.105 |
| 1935-39 | 0.837 | 0.147 | 0.627 | 1.117 |
| 1930-34 | 0.595 | 0.168 | 0.429 | 0.827 |
| 1900-29 | 0.518 | 0.170 | 0.371 | 0.722 |
| Time | 1.095 | 0.023 | 1.048 | 1.145 |
| Cohort*time (ref= 1950-54) | |  |  |  |
| 1945-49 | 0.987 | 0.014 | 0.959 | 1.015 |
| 1940-44 | 0.973 | 0.015 | 0.944 | 1.003 |
| 1935-39 | 0.951 | 0.017 | 0.920 | 0.983 |
| 1930-34 | 0.967 | 0.020 | 0.929 | 1.007 |
| 1900-29 | 0.954 | 0.023 | 0.912 | 0.997 |
| Gender*time | 0.994 | 0.011 | 0.973 | 1.016 |
| Cohort*gender (ref= 1950-54) | |  |  |  |
| 1945-49 | 0.834 | 0.139 | 0.636 | 1.095 |
| 1940-44 | 0.847 | 0.146 | 0.636 | 1.127 |
| 1935-39 | 0.743 | 0.153 | 0.550 | 1.002 |
| 1930-34 | 0.587 | 0.177 | 0.415 | 0.830 |
| 1900-29 | 0.469 | 0.180 | 0.330 | 0.666 |
| Time*time | 0.984 | 0.002 | 0.980 | 0.988 |
| Education (ref=primary) |  |  |  |  |
| Secondary | 1.196 | 0.053 | 1.078 | 1.327 |
| Tertiary | 1.003 | 0.069 | 0.877 | 1.149 |
| Self-rated health (ref=Excellent) | |  |  |  |
| Very good | 1.051 | 0.080 | 0.898 | 1.230 |
| Good | 1.110 | 0.076 | 0.956 | 1.289 |
| Fair | 1.008 | 0.085 | 0.853 | 1.191 |
| Poor | 0.663 | 0.117 | 0.527 | 0.835 |
| Employed (ref=no) | 0.529 | 0.063 | 0.467 | 0.599 |
| Chronic condition | 1.023 | 0.016 | 0.991 | 1.057 |
| Intercept | 0.020 | 0.146 | 0.015 | 0.027 |
| **Random Effects** |  |  |  |  |
| Wave | 0.011 |  | 0.006 | 0.018 |
| Intercept | 2.001 |  | 1.775 | 2.255 |
| No. of observations: 78,607 |  | No. of individuals: 25,480 | | |

**Supplementary Table 3:** Mixed effects logistic regression for informal caregiving (all types), by care regime (underlying model for Figure 3) (LCI, lower confidence interval; UCI, upper confidence interval)

| **Continental care regime** | Odds-Ratio | P-value | LCI | UCI |
| --- | --- | --- | --- | --- |
| **Fixed Effects** |  |  |  |  |
| Sex (ref=male) | 1.294 | 0.021 | 1.040 | 1.610 |
| Cohort (ref= 1950-54) |  |  |  |  |
| 1945-49 | 0.910 | 0.442 | 0.716 | 1.157 |
| 1940-44 | 0.865 | 0.261 | 0.673 | 1.113 |
| 1935-39 | 0.922 | 0.548 | 0.708 | 1.201 |
| 1930-34 | 0.566 | 0.000 | 0.420 | 0.764 |
| 1900-29 | 0.492 | 0.000 | 0.363 | 0.667 |
| Time | 0.982 | 0.387 | 0.943 | 1.023 |
| Cohort*time (ref= 1950-54) | |  |  |  |
| 1945-49 | 0.975 | 0.085 | 0.947 | 1.003 |
| 1940-44 | 0.961 | 0.010 | 0.933 | 0.990 |
| 1935-39 | 0.941 | 0.000 | 0.911 | 0.972 |
| 1930-34 | 0.955 | 0.011 | 0.922 | 0.989 |
| 1900-29 | 0.936 | 0.001 | 0.900 | 0.974 |
| Gender*time | 0.999 | 0.891 | 0.979 | 1.018 |
| Cohort*gender (ref= 1950-54) | |  |  |  |
| 1945-49 | 0.965 | 0.794 | 0.740 | 1.259 |
| 1940-44 | 0.845 | 0.227 | 0.643 | 1.111 |
| 1935-39 | 0.709 | 0.017 | 0.534 | 0.941 |
| 1930-34 | 0.680 | 0.022 | 0.489 | 0.946 |
| 1900-29 | 0.480 | 0.000 | 0.345 | 0.668 |
| Time*time | 1.001 | 0.738 | 0.997 | 1.004 |
| Education (ref=Primary) |  |  |  |  |
| Secondary | 1.509 | 0.000 | 1.351 | 1.687 |
| Tertiary | 1.895 | 0.000 | 1.657 | 2.169 |
| Self-rated health (ref=Excellent) | |  |  |  |
| Very good | 0.873 | 0.097 | 0.744 | 1.025 |
| Good | 0.801 | 0.004 | 0.689 | 0.932 |
| Fair | 0.708 | 0.000 | 0.598 | 0.838 |
| Poor | 0.483 | 0.000 | 0.389 | 0.599 |
| Partner in household (ref=no) | 0.798 | 0.000 | 0.721 | 0.884 |
| Employed (ref=no) | 0.935 | 0.278 | 0.828 | 1.056 |
| Chronic condition | 1.062 | 0.000 | 1.029 | 1.096 |
| Intercept | 0.623 | 0.000 | 0.480 | 0.808 |
| **Random Effects** |  |  |  |  |
| Wave | 0.010 |  | 0.007 | 0.014 |
| Intercept | 0.972 |  | 0.841 | 1.125 |
| No. of observations: 39,562 |  | No. of individuals: 12,840 | | |

| Southern care regime | Odds-Ratio | P-value | LCI | UCI |
| --- | --- | --- | --- | --- |
| **Fixed Effects** |  |  |  |  |
| Sex (ref=male) | 1.687 | 0.001 | 1.224 | 2.326 |
| Cohort (ref= 1950-54) |  |  |  |  |
| 1945-49 | 0.868 | 0.440 | 0.605 | 1.244 |
| 1940-44 | 0.952 | 0.788 | 0.663 | 1.365 |
| 1935-39 | 0.562 | 0.003 | 0.382 | 0.827 |
| 1930-34 | 0.566 | 0.005 | 0.379 | 0.844 |
| 1900-29 | 0.446 | 0.000 | 0.293 | 0.678 |
| Time | 1.088 | 0.002 | 1.032 | 1.147 |
| Cohort*time (ref= 1950-54) | |  |  |  |
| 1945-49 | 0.997 | 0.865 | 0.961 | 1.034 |
| 1940-44 | 0.954 | 0.013 | 0.920 | 0.990 |
| 1935-39 | 1.006 | 0.777 | 0.967 | 1.046 |
| 1930-34 | 1.008 | 0.717 | 0.964 | 1.055 |
| 1900-29 | 1.024 | 0.366 | 0.973 | 1.077 |
| Gender*time | 1.007 | 0.603 | 0.982 | 1.031 |
| Cohort*gender (ref= 1950-54) | |  |  |  |
| 1945-49 | 1.006 | 0.974 | 0.685 | 1.478 |
| 1940-44 | 0.921 | 0.678 | 0.624 | 1.359 |
| 1935-39 | 0.891 | 0.571 | 0.598 | 1.328 |
| 1930-34 | 0.764 | 0.211 | 0.501 | 1.164 |
| 1900-29 | 0.561 | 0.012 | 0.358 | 0.878 |
| Time*time | 0.987 | 0.000 | 0.983 | 0.991 |
| Education (ref=Primary) |  |  |  |  |
| Secondary | 1.355 | 0.000 | 1.185 | 1.549 |
| Tertiary | 1.606 | 0.000 | 1.284 | 2.009 |
| Self-rated health (ref=Excellent) | |  |  |  |
| Very good | 0.907 | 0.436 | 0.709 | 1.160 |
| Good | 0.921 | 0.484 | 0.732 | 1.159 |
| Fair | 0.843 | 0.178 | 0.657 | 1.081 |
| Poor | 0.749 | 0.050 | 0.561 | 1.000 |
| Partner in household (ref=no) | 0.662 | 0.000 | 0.573 | 0.766 |
| Employed (ref=no) | 0.831 | 0.061 | 0.685 | 1.009 |
| Chronic condition | 1.085 | 0.000 | 1.044 | 1.127 |
| Intercept | 0.298 | 0.000 | 0.200 | 0.445 |
| **Random Effects** |  |  |  |  |
| Wave | 0.008 |  | 0.004 | 0.0138 |
| Intercept | 1.215 |  | 1.026 | 1.44 |
| No. of observations: 23,111 |  | No. of individuals: 7,711 | | |

| **Nordic care regime** | Odds-Ratio | P-value | LCI | UCI |
| --- | --- | --- | --- | --- |
| **Fixed Effects** |  |  |  |  |
| Sex (ref=male) | 1.064 | 0.638 | 0.821 | 1.380 |
| Cohort (ref= 1950-54) |  |  |  |  |
| 1945-49 | 0.865 | 0.323 | 0.649 | 1.153 |
| 1940-44 | 0.600 | 0.001 | 0.446 | 0.806 |
| 1935-39 | 0.451 | 0.000 | 0.326 | 0.626 |
| 1930-34 | 0.450 | 0.000 | 0.312 | 0.649 |
| 1900-29 | 0.208 | 0.000 | 0.146 | 0.296 |
| Time | 0.971 | 0.211 | 0.927 | 1.017 |
| Cohort*time (ref= 1950-54) | |  |  |  |
| 1945-49 | 1.018 | 0.305 | 0.984 | 1.052 |
| 1940-44 | 1.021 | 0.242 | 0.986 | 1.056 |
| 1935-39 | 1.008 | 0.692 | 0.971 | 1.046 |
| 1930-34 | 0.972 | 0.193 | 0.932 | 1.014 |
| 1900-29 | 0.972 | 0.234 | 0.928 | 1.018 |
| Gender*time | 0.993 | 0.551 | 0.972 | 1.016 |
| Cohort*gender (ref= 1950-54) | |  |  |  |
| 1945-49 | 0.932 | 0.672 | 0.674 | 1.289 |
| 1940-44 | 0.915 | 0.598 | 0.658 | 1.273 |
| 1935-39 | 0.868 | 0.433 | 0.609 | 1.236 |
| 1930-34 | 0.774 | 0.204 | 0.521 | 1.149 |
| 1900-29 | 0.664 | 0.033 | 0.455 | 0.968 |
| Time*time | 0.997 | 0.131 | 0.994 | 1.001 |
| Education (ref=Primary) |  |  |  |  |
| Secondary | 1.198 | 0.012 | 1.041 | 1.379 |
| Tertiary | 1.292 | 0.001 | 1.105 | 1.511 |
| Self-rated health (ref=Excellent) | |  |  |  |
| Very good | 1.036 | 0.599 | 0.907 | 1.183 |
| Good | 0.913 | 0.209 | 0.792 | 1.052 |
| Fair | 0.664 | 0.000 | 0.560 | 0.788 |
| Poor | 0.483 | 0.000 | 0.377 | 0.617 |
| Partner in household (ref=no) | 1.002 | 0.973 | 0.892 | 1.126 |
| Employed (ref=no) | 0.956 | 0.528 | 0.830 | 1.100 |
| Chronic condition | 1.038 | 0.057 | 0.999 | 1.078 |
| Intercept | 1.510 | 0.007 | 1.117 | 2.043 |
| **Random Effects** |  |  |  |  |
| Wave | 0.011 |  | 0.007 | 0.016 |
| Intercept | 1.047 |  | 0.885 | 1.238 |
| No. of observations: 15,934 |  | No. of individuals: 4,929 | | |

**Supplementary Table 4:** Predicted probabilities for informal caregiving (all types) by cohort, age and sex (Figure 1). Pooled sample, adjusted for control variables (PP, predicted probability; LCI, lower confidence interval; UCI, upper confidence interval). Weighted values.

|  |  |  | Men | | | Women | | |
| --- | --- | --- | --- | --- | --- | --- | --- | --- |
| Cohort | Birth years | Age | PP | LCI | UCI | PP | LCI | UCI |
| 1 | 1950-54 | 52 | 0.368 | 0.338 | 0.398 | 0.434 | 0.408 | 0.460 |
|  | 1950-54 | 53 | 0.372 | 0.344 | 0.400 | 0.439 | 0.415 | 0.462 |
|  | 1950-54 | 54 | 0.374 | 0.347 | 0.401 | 0.441 | 0.419 | 0.463 |
|  | 1950-54 | 55 | 0.374 | 0.348 | 0.400 | 0.442 | 0.420 | 0.463 |
|  | 1950-54 | 59 | 0.358 | 0.334 | 0.382 | 0.424 | 0.403 | 0.445 |
|  | 1950-54 | 60 | 0.350 | 0.326 | 0.374 | 0.415 | 0.394 | 0.436 |
|  | 1950-54 | 61 | 0.341 | 0.317 | 0.365 | 0.405 | 0.383 | 0.426 |
|  | 1950-54 | 63 | 0.319 | 0.293 | 0.345 | 0.380 | 0.356 | 0.405 |
| 2 | 1945-49 | 57 | 0.346 | 0.320 | 0.372 | 0.410 | 0.387 | 0.434 |
|  | 1945-49 | 58 | 0.347 | 0.324 | 0.371 | 0.412 | 0.391 | 0.433 |
|  | 1945-49 | 59 | 0.346 | 0.325 | 0.368 | 0.412 | 0.392 | 0.431 |
|  | 1945-49 | 60 | 0.344 | 0.323 | 0.365 | 0.409 | 0.390 | 0.428 |
|  | 1945-49 | 64 | 0.320 | 0.300 | 0.339 | 0.382 | 0.363 | 0.402 |
|  | 1945-49 | 65 | 0.310 | 0.291 | 0.330 | 0.372 | 0.352 | 0.391 |
|  | 1945-49 | 66 | 0.300 | 0.280 | 0.320 | 0.360 | 0.340 | 0.380 |
|  | 1945-49 | 68 | 0.276 | 0.254 | 0.299 | 0.333 | 0.310 | 0.356 |
| 3 | 1940-44 | 62 | 0.347 | 0.323 | 0.371 | 0.389 | 0.365 | 0.413 |
|  | 1940-44 | 63 | 0.344 | 0.322 | 0.366 | 0.386 | 0.364 | 0.408 |
|  | 1940-44 | 64 | 0.339 | 0.319 | 0.359 | 0.381 | 0.361 | 0.402 |
|  | 1940-44 | 65 | 0.333 | 0.313 | 0.352 | 0.375 | 0.355 | 0.394 |
|  | 1940-44 | 69 | 0.294 | 0.275 | 0.313 | 0.334 | 0.314 | 0.354 |
|  | 1940-44 | 70 | 0.282 | 0.262 | 0.301 | 0.321 | 0.301 | 0.341 |
|  | 1940-44 | 71 | 0.269 | 0.249 | 0.288 | 0.307 | 0.286 | 0.327 |
|  | 1940-44 | 73 | 0.241 | 0.220 | 0.263 | 0.277 | 0.254 | 0.300 |
| 4 | 1935-39 | 67 | 0.325 | 0.300 | 0.350 | 0.342 | 0.317 | 0.366 |
|  | 1935-39 | 68 | 0.322 | 0.300 | 0.345 | 0.340 | 0.318 | 0.361 |
|  | 1935-39 | 69 | 0.318 | 0.297 | 0.339 | 0.336 | 0.315 | 0.356 |
|  | 1935-39 | 70 | 0.313 | 0.292 | 0.333 | 0.331 | 0.311 | 0.350 |
|  | 1935-39 | 74 | 0.278 | 0.258 | 0.298 | 0.296 | 0.276 | 0.316 |
|  | 1935-39 | 75 | 0.267 | 0.247 | 0.288 | 0.285 | 0.265 | 0.305 |
|  | 1935-39 | 76 | 0.255 | 0.234 | 0.276 | 0.273 | 0.252 | 0.294 |
|  | 1935-39 | 78 | 0.230 | 0.207 | 0.253 | 0.247 | 0.224 | 0.270 |
| 5 | 1930-34 | 72 | 0.270 | 0.244 | 0.296 | 0.278 | 0.250 | 0.306 |
|  | 1930-34 | 73 | 0.270 | 0.245 | 0.294 | 0.278 | 0.253 | 0.304 |
|  | 1930-34 | 74 | 0.268 | 0.245 | 0.291 | 0.277 | 0.253 | 0.301 |
|  | 1930-34 | 75 | 0.265 | 0.243 | 0.287 | 0.274 | 0.252 | 0.297 |
|  | 1930-34 | 79 | 0.241 | 0.219 | 0.263 | 0.251 | 0.230 | 0.272 |
|  | 1930-34 | 80 | 0.233 | 0.210 | 0.256 | 0.243 | 0.222 | 0.264 |
|  | 1930-34 | 81 | 0.224 | 0.200 | 0.248 | 0.234 | 0.212 | 0.256 |
|  | 1930-34 | 83 | 0.204 | 0.178 | 0.230 | 0.214 | 0.190 | 0.239 |
| 6 | 1900-29 | 80 | 0.240 | 0.215 | 0.266 | 0.198 | 0.176 | 0.219 |
|  | 1900-29 | 81 | 0.239 | 0.215 | 0.263 | 0.197 | 0.177 | 0.217 |
|  | 1900-29 | 82 | 0.237 | 0.214 | 0.259 | 0.196 | 0.177 | 0.214 |
|  | 1900-29 | 83 | 0.234 | 0.212 | 0.256 | 0.193 | 0.176 | 0.211 |
|  | 1900-29 | 86 | 0.211 | 0.187 | 0.235 | 0.176 | 0.157 | 0.195 |
|  | 1900-29 | 87 | 0.203 | 0.179 | 0.228 | 0.170 | 0.150 | 0.190 |
|  | 1900-29 | 88 | 0.195 | 0.169 | 0.221 | 0.164 | 0.143 | 0.184 |
|  | 1900-29 | 89 | 0.177 | 0.149 | 0.205 | 0.149 | 0.126 | 0.173 |

**Supplementary Table 5a:** Predicted probabilities for care outside the household by cohort, age and sex (Figure 2a). Pooled sample, adjusted for control variables (PP, predicted probability; LCI, lower confidence interval; UCI, upper confidence interval). Weighted values.

|  |  |  | Men | | | Women | | |
| --- | --- | --- | --- | --- | --- | --- | --- | --- |
| Cohort | Birth years | Age | PP | LCI | UCI | PP | LCI | UCI |
| 1 | 1950-54 | 52 | 0.354 | 0.324 | 0.384 | 0.403 | 0.378 | 0.428 |
|  | 1950-54 | 53 | 0.350 | 0.322 | 0.378 | 0.400 | 0.378 | 0.423 |
|  | 1950-54 | 54 | 0.346 | 0.319 | 0.372 | 0.396 | 0.375 | 0.417 |
|  | 1950-54 | 55 | 0.340 | 0.315 | 0.366 | 0.391 | 0.371 | 0.412 |
|  | 1950-54 | 59 | 0.312 | 0.288 | 0.335 | 0.361 | 0.342 | 0.381 |
|  | 1950-54 | 60 | 0.303 | 0.280 | 0.327 | 0.352 | 0.332 | 0.372 |
|  | 1950-54 | 61 | 0.294 | 0.271 | 0.317 | 0.342 | 0.322 | 0.362 |
|  | 1950-54 | 63 | 0.275 | 0.250 | 0.300 | 0.321 | 0.298 | 0.344 |
| 2 | 1945-49 | 57 | 0.334 | 0.309 | 0.360 | 0.383 | 0.360 | 0.407 |
|  | 1945-49 | 58 | 0.328 | 0.305 | 0.351 | 0.377 | 0.357 | 0.398 |
|  | 1945-49 | 59 | 0.321 | 0.300 | 0.343 | 0.370 | 0.351 | 0.390 |
|  | 1945-49 | 60 | 0.314 | 0.294 | 0.334 | 0.363 | 0.344 | 0.381 |
|  | 1945-49 | 64 | 0.277 | 0.259 | 0.296 | 0.324 | 0.306 | 0.343 |
|  | 1945-49 | 65 | 0.267 | 0.249 | 0.286 | 0.313 | 0.295 | 0.332 |
|  | 1945-49 | 66 | 0.257 | 0.238 | 0.276 | 0.302 | 0.283 | 0.321 |
|  | 1945-49 | 68 | 0.235 | 0.214 | 0.257 | 0.278 | 0.256 | 0.300 |
| 3 | 1940-44 | 62 | 0.335 | 0.311 | 0.359 | 0.348 | 0.324 | 0.371 |
|  | 1940-44 | 63 | 0.325 | 0.303 | 0.346 | 0.338 | 0.317 | 0.359 |
|  | 1940-44 | 64 | 0.314 | 0.294 | 0.334 | 0.328 | 0.308 | 0.347 |
|  | 1940-44 | 65 | 0.303 | 0.284 | 0.322 | 0.317 | 0.298 | 0.336 |
|  | 1940-44 | 69 | 0.254 | 0.236 | 0.272 | 0.268 | 0.250 | 0.287 |
|  | 1940-44 | 70 | 0.242 | 0.223 | 0.260 | 0.256 | 0.237 | 0.274 |
|  | 1940-44 | 71 | 0.229 | 0.210 | 0.248 | 0.243 | 0.224 | 0.262 |
|  | 1940-44 | 73 | 0.204 | 0.184 | 0.225 | 0.218 | 0.197 | 0.239 |
| 4 | 1935-39 | 67 | 0.317 | 0.291 | 0.342 | 0.308 | 0.285 | 0.332 |
|  | 1935-39 | 68 | 0.303 | 0.280 | 0.326 | 0.295 | 0.274 | 0.316 |
|  | 1935-39 | 69 | 0.289 | 0.268 | 0.310 | 0.282 | 0.262 | 0.301 |
|  | 1935-39 | 70 | 0.274 | 0.254 | 0.294 | 0.268 | 0.249 | 0.286 |
|  | 1935-39 | 74 | 0.216 | 0.198 | 0.234 | 0.212 | 0.194 | 0.230 |
|  | 1935-39 | 75 | 0.202 | 0.184 | 0.220 | 0.199 | 0.181 | 0.217 |
|  | 1935-39 | 76 | 0.188 | 0.170 | 0.207 | 0.186 | 0.167 | 0.204 |
|  | 1935-39 | 78 | 0.162 | 0.143 | 0.182 | 0.161 | 0.141 | 0.181 |
| 5 | 1930-34 | 72 | 0.240 | 0.214 | 0.267 | 0.229 | 0.203 | 0.256 |
|  | 1930-34 | 73 | 0.229 | 0.205 | 0.252 | 0.219 | 0.195 | 0.243 |
|  | 1930-34 | 74 | 0.217 | 0.195 | 0.239 | 0.208 | 0.186 | 0.229 |
|  | 1930-34 | 75 | 0.205 | 0.185 | 0.226 | 0.197 | 0.176 | 0.217 |
|  | 1930-34 | 79 | 0.159 | 0.140 | 0.178 | 0.153 | 0.136 | 0.171 |
|  | 1930-34 | 80 | 0.148 | 0.129 | 0.167 | 0.143 | 0.126 | 0.161 |
|  | 1930-34 | 81 | 0.138 | 0.119 | 0.157 | 0.133 | 0.116 | 0.151 |
|  | 1930-34 | 83 | 0.118 | 0.098 | 0.138 | 0.115 | 0.097 | 0.134 |
| 6 | 1900-29 | 80 | 0.181 | 0.156 | 0.206 | 0.147 | 0.127 | 0.168 |
|  | 1900-29 | 81 | 0.169 | 0.147 | 0.192 | 0.138 | 0.120 | 0.155 |
|  | 1900-29 | 82 | 0.158 | 0.137 | 0.179 | 0.128 | 0.112 | 0.144 |
|  | 1900-29 | 83 | 0.147 | 0.127 | 0.166 | 0.119 | 0.104 | 0.134 |
|  | 1900-29 | 86 | 0.106 | 0.089 | 0.124 | 0.086 | 0.073 | 0.100 |
|  | 1900-29 | 87 | 0.098 | 0.080 | 0.116 | 0.079 | 0.066 | 0.093 |
|  | 1900-29 | 88 | 0.089 | 0.071 | 0.107 | 0.073 | 0.059 | 0.087 |
|  | 1900-29 | 89 | 0.075 | 0.056 | 0.093 | 0.061 | 0.047 | 0.075 |

**Supplementary Table 5b:** Predicted probabilities for care inside the household by cohort, age and sex (Figure 2b). Pooled sample, adjusted for control variables (PP, predicted probability; LCI, lower confidence interval; UCI, upper confidence interval). Weighted values.

|  |  |  | Men | | | Women | | |
| --- | --- | --- | --- | --- | --- | --- | --- | --- |
| Cohort | Birth years | Age | PP | LCI | UCI | PP | LCI | UCI |
| 1 | 1950-54 | 52 | 0.048 | 0.035 | 0.062 | 0.066 | 0.052 | 0.079 |
|  | 1950-54 | 53 | 0.058 | 0.044 | 0.072 | 0.078 | 0.064 | 0.092 |
|  | 1950-54 | 54 | 0.067 | 0.053 | 0.082 | 0.089 | 0.075 | 0.104 |
|  | 1950-54 | 55 | 0.076 | 0.061 | 0.092 | 0.100 | 0.085 | 0.114 |
|  | 1950-54 | 59 | 0.096 | 0.079 | 0.112 | 0.120 | 0.104 | 0.137 |
|  | 1950-54 | 60 | 0.095 | 0.079 | 0.112 | 0.119 | 0.102 | 0.135 |
|  | 1950-54 | 61 | 0.093 | 0.076 | 0.109 | 0.115 | 0.098 | 0.132 |
|  | 1950-54 | 63 | 0.082 | 0.066 | 0.099 | 0.101 | 0.083 | 0.119 |
| 2 | 1945-49 | 57 | 0.043 | 0.033 | 0.053 | 0.064 | 0.052 | 0.076 |
|  | 1945-49 | 58 | 0.051 | 0.040 | 0.062 | 0.075 | 0.063 | 0.087 |
|  | 1945-49 | 59 | 0.059 | 0.048 | 0.071 | 0.086 | 0.074 | 0.098 |
|  | 1945-49 | 60 | 0.067 | 0.055 | 0.079 | 0.096 | 0.083 | 0.108 |
|  | 1945-49 | 64 | 0.083 | 0.071 | 0.096 | 0.114 | 0.099 | 0.128 |
|  | 1945-49 | 65 | 0.083 | 0.070 | 0.096 | 0.112 | 0.098 | 0.126 |
|  | 1945-49 | 66 | 0.080 | 0.068 | 0.093 | 0.108 | 0.094 | 0.123 |
|  | 1945-49 | 68 | 0.071 | 0.058 | 0.084 | 0.094 | 0.079 | 0.109 |
| 3 | 1940-44 | 62 | 0.047 | 0.036 | 0.058 | 0.076 | 0.063 | 0.090 |
|  | 1940-44 | 63 | 0.055 | 0.044 | 0.066 | 0.088 | 0.074 | 0.102 |
|  | 1940-44 | 64 | 0.063 | 0.051 | 0.074 | 0.099 | 0.085 | 0.113 |
|  | 1940-44 | 65 | 0.070 | 0.058 | 0.082 | 0.109 | 0.094 | 0.123 |
|  | 1940-44 | 69 | 0.083 | 0.070 | 0.096 | 0.123 | 0.106 | 0.140 |
|  | 1940-44 | 70 | 0.082 | 0.068 | 0.095 | 0.120 | 0.103 | 0.137 |
|  | 1940-44 | 71 | 0.078 | 0.065 | 0.091 | 0.114 | 0.097 | 0.131 |
|  | 1940-44 | 73 | 0.067 | 0.054 | 0.081 | 0.098 | 0.080 | 0.115 |
| 4 | 1935-39 | 67 | 0.037 | 0.028 | 0.046 | 0.063 | 0.050 | 0.075 |
|  | 1935-39 | 68 | 0.047 | 0.037 | 0.056 | 0.077 | 0.064 | 0.091 |
|  | 1935-39 | 69 | 0.057 | 0.046 | 0.068 | 0.093 | 0.079 | 0.107 |
|  | 1935-39 | 70 | 0.068 | 0.056 | 0.079 | 0.108 | 0.092 | 0.123 |
|  | 1935-39 | 74 | 0.100 | 0.085 | 0.115 | 0.148 | 0.129 | 0.168 |
|  | 1935-39 | 75 | 0.103 | 0.087 | 0.119 | 0.151 | 0.131 | 0.171 |
|  | 1935-39 | 76 | 0.104 | 0.087 | 0.121 | 0.151 | 0.130 | 0.172 |
|  | 1935-39 | 78 | 0.099 | 0.081 | 0.117 | 0.142 | 0.119 | 0.164 |
| 5 | 1930-34 | 72 | 0.055 | 0.042 | 0.067 | 0.084 | 0.067 | 0.102 |
|  | 1930-34 | 73 | 0.067 | 0.054 | 0.080 | 0.101 | 0.083 | 0.120 |
|  | 1930-34 | 74 | 0.080 | 0.066 | 0.094 | 0.118 | 0.100 | 0.137 |
|  | 1930-34 | 75 | 0.092 | 0.077 | 0.107 | 0.134 | 0.115 | 0.154 |
|  | 1930-34 | 79 | 0.125 | 0.105 | 0.144 | 0.172 | 0.149 | 0.195 |
|  | 1930-34 | 80 | 0.127 | 0.106 | 0.147 | 0.173 | 0.150 | 0.197 |
|  | 1930-34 | 81 | 0.126 | 0.104 | 0.147 | 0.170 | 0.146 | 0.195 |
|  | 1930-34 | 83 | 0.116 | 0.093 | 0.139 | 0.156 | 0.129 | 0.183 |
| 6 | 1900-29 | 80 | 0.087 | 0.071 | 0.103 | 0.093 | 0.076 | 0.111 |
|  | 1900-29 | 81 | 0.103 | 0.087 | 0.120 | 0.109 | 0.091 | 0.127 |
|  | 1900-29 | 82 | 0.118 | 0.101 | 0.136 | 0.124 | 0.106 | 0.143 |
|  | 1900-29 | 83 | 0.132 | 0.114 | 0.150 | 0.138 | 0.118 | 0.158 |
|  | 1900-29 | 86 | 0.160 | 0.136 | 0.183 | 0.164 | 0.139 | 0.189 |
|  | 1900-29 | 87 | 0.158 | 0.133 | 0.184 | 0.162 | 0.136 | 0.188 |
|  | 1900-29 | 88 | 0.154 | 0.128 | 0.180 | 0.157 | 0.129 | 0.185 |
|  | 1900-29 | 89 | 0.137 | 0.108 | 0.166 | 0.139 | 0.108 | 0.169 |

**Supplementary Table 5c:** Predicted probabilities for intensive care by cohort, age and sex (Figure 2c). Pooled sample, adjusted for control variables (PP, predicted probability; LCI, lower confidence interval; UCI, upper confidence interval). Weighted values.

|  |  |  | Men | | | Women | | |
| --- | --- | --- | --- | --- | --- | --- | --- | --- |
| Cohort | Birth years | Age | PP | LCI | UCI | PP | LCI | UCI |
| 1 | 1950-54 | 52 | 0.046 | 0.037 | 0.054 | 0.096 | 0.085 | 0.108 |
|  | 1950-54 | 53 | 0.049 | 0.041 | 0.057 | 0.102 | 0.091 | 0.112 |
|  | 1950-54 | 54 | 0.051 | 0.043 | 0.059 | 0.105 | 0.095 | 0.115 |
|  | 1950-54 | 55 | 0.052 | 0.044 | 0.060 | 0.107 | 0.097 | 0.117 |
|  | 1950-54 | 59 | 0.047 | 0.040 | 0.054 | 0.094 | 0.084 | 0.103 |
|  | 1950-54 | 60 | 0.044 | 0.037 | 0.050 | 0.086 | 0.077 | 0.095 |
|  | 1950-54 | 61 | 0.040 | 0.033 | 0.046 | 0.078 | 0.070 | 0.087 |
|  | 1950-54 | 63 | 0.031 | 0.025 | 0.037 | 0.061 | 0.052 | 0.069 |
| 2 | 1945-49 | 57 | 0.045 | 0.037 | 0.053 | 0.083 | 0.073 | 0.093 |
|  | 1945-49 | 58 | 0.048 | 0.040 | 0.055 | 0.087 | 0.078 | 0.096 |
|  | 1945-49 | 59 | 0.049 | 0.042 | 0.056 | 0.089 | 0.081 | 0.097 |
|  | 1945-49 | 60 | 0.050 | 0.043 | 0.057 | 0.089 | 0.081 | 0.097 |
|  | 1945-49 | 64 | 0.043 | 0.037 | 0.049 | 0.075 | 0.068 | 0.083 |
|  | 1945-49 | 65 | 0.039 | 0.034 | 0.045 | 0.069 | 0.062 | 0.076 |
|  | 1945-49 | 66 | 0.035 | 0.030 | 0.041 | 0.062 | 0.055 | 0.069 |
|  | 1945-49 | 68 | 0.027 | 0.022 | 0.032 | 0.047 | 0.040 | 0.054 |
| 3 | 1940-44 | 62 | 0.039 | 0.033 | 0.046 | 0.074 | 0.065 | 0.083 |
|  | 1940-44 | 63 | 0.041 | 0.035 | 0.048 | 0.077 | 0.068 | 0.085 |
|  | 1940-44 | 64 | 0.042 | 0.036 | 0.048 | 0.078 | 0.070 | 0.086 |
|  | 1940-44 | 65 | 0.042 | 0.036 | 0.048 | 0.078 | 0.070 | 0.085 |
|  | 1940-44 | 69 | 0.035 | 0.029 | 0.040 | 0.063 | 0.055 | 0.070 |
|  | 1940-44 | 70 | 0.031 | 0.026 | 0.037 | 0.057 | 0.050 | 0.064 |
|  | 1940-44 | 71 | 0.028 | 0.023 | 0.033 | 0.050 | 0.043 | 0.057 |
|  | 1940-44 | 73 | 0.021 | 0.016 | 0.025 | 0.037 | 0.031 | 0.043 |
| 4 | 1935-39 | 67 | 0.039 | 0.032 | 0.046 | 0.067 | 0.057 | 0.076 |
|  | 1935-39 | 68 | 0.040 | 0.034 | 0.047 | 0.068 | 0.059 | 0.076 |
|  | 1935-39 | 69 | 0.040 | 0.034 | 0.047 | 0.068 | 0.060 | 0.076 |
|  | 1935-39 | 70 | 0.040 | 0.034 | 0.046 | 0.066 | 0.059 | 0.074 |
|  | 1935-39 | 74 | 0.030 | 0.025 | 0.036 | 0.050 | 0.043 | 0.057 |
|  | 1935-39 | 75 | 0.027 | 0.022 | 0.032 | 0.044 | 0.037 | 0.051 |
|  | 1935-39 | 76 | 0.023 | 0.019 | 0.028 | 0.038 | 0.032 | 0.045 |
|  | 1935-39 | 78 | 0.017 | 0.012 | 0.021 | 0.027 | 0.021 | 0.033 |
| 5 | 1930-34 | 72 | 0.029 | 0.023 | 0.036 | 0.042 | 0.033 | 0.050 |
|  | 1930-34 | 73 | 0.030 | 0.024 | 0.037 | 0.043 | 0.035 | 0.050 |
|  | 1930-34 | 74 | 0.031 | 0.025 | 0.037 | 0.043 | 0.036 | 0.051 |
|  | 1930-34 | 75 | 0.031 | 0.025 | 0.037 | 0.043 | 0.036 | 0.050 |
|  | 1930-34 | 79 | 0.025 | 0.019 | 0.031 | 0.034 | 0.028 | 0.040 |
|  | 1930-34 | 80 | 0.022 | 0.017 | 0.028 | 0.031 | 0.024 | 0.037 |
|  | 1930-34 | 81 | 0.020 | 0.015 | 0.025 | 0.027 | 0.021 | 0.033 |
|  | 1930-34 | 83 | 0.015 | 0.010 | 0.019 | 0.020 | 0.014 | 0.025 |
| 6 | 1900-29 | 80 | 0.026 | 0.020 | 0.032 | 0.030 | 0.024 | 0.036 |
|  | 1900-29 | 81 | 0.027 | 0.021 | 0.032 | 0.031 | 0.026 | 0.036 |
|  | 1900-29 | 82 | 0.027 | 0.021 | 0.032 | 0.031 | 0.026 | 0.036 |
|  | 1900-29 | 83 | 0.026 | 0.021 | 0.032 | 0.030 | 0.026 | 0.035 |
|  | 1900-29 | 86 | 0.020 | 0.015 | 0.025 | 0.023 | 0.018 | 0.028 |
|  | 1900-29 | 87 | 0.018 | 0.013 | 0.023 | 0.020 | 0.016 | 0.025 |
|  | 1900-29 | 88 | 0.016 | 0.011 | 0.020 | 0.018 | 0.013 | 0.022 |
|  | 1900-29 | 89 | 0.011 | 0.007 | 0.015 | 0.012 | 0.008 | 0.017 |

**Supplementary Table 6**: Predicted probabilities for informal caregiving (all types) by cohort, age, sex and care regime (Figure 3). Adjusted for control variables (PP, predicted probability; LCI, lower confidence interval; UCI, upper confidence interval). Weighted values.

| Continental care regime | | | | | | | | | |
| --- | --- | --- | --- | --- | --- | --- | --- | --- | --- |
|  |  |  | Men | | | Women | | | |
| Cohort | Birth years | Age | PP | LCI | UCI | PP | LCI | UCI | |
| 1 | 1950-54 | 52 | 0.428 | 0.386 | 0.469 | 0.480 | 0.444 | 0.515 |  |
|  | 1950-54 | 53 | 0.424 | 0.386 | 0.462 | 0.476 | 0.444 | 0.508 |  |
|  | 1950-54 | 54 | 0.421 | 0.385 | 0.457 | 0.473 | 0.443 | 0.502 |  |
|  | 1950-54 | 55 | 0.419 | 0.384 | 0.453 | 0.469 | 0.441 | 0.497 |  |
|  | 1950-54 | 59 | 0.413 | 0.381 | 0.446 | 0.460 | 0.433 | 0.487 |  |
|  | 1950-54 | 60 | 0.413 | 0.380 | 0.446 | 0.459 | 0.431 | 0.487 |  |
|  | 1950-54 | 61 | 0.413 | 0.380 | 0.447 | 0.458 | 0.429 | 0.487 |  |
|  | 1950-54 | 63 | 0.414 | 0.376 | 0.452 | 0.457 | 0.423 | 0.491 |  |
| 2 | 1945-49 | 57 | 0.409 | 0.373 | 0.444 | 0.453 | 0.420 | 0.487 |  |
|  | 1945-49 | 58 | 0.400 | 0.369 | 0.432 | 0.444 | 0.415 | 0.474 |  |
|  | 1945-49 | 59 | 0.393 | 0.364 | 0.421 | 0.436 | 0.408 | 0.464 |  |
|  | 1945-49 | 60 | 0.385 | 0.358 | 0.413 | 0.428 | 0.401 | 0.455 |  |
|  | 1945-49 | 64 | 0.363 | 0.337 | 0.390 | 0.402 | 0.374 | 0.430 |  |
|  | 1945-49 | 65 | 0.359 | 0.332 | 0.387 | 0.397 | 0.368 | 0.426 |  |
|  | 1945-49 | 66 | 0.356 | 0.327 | 0.385 | 0.393 | 0.362 | 0.423 |  |
|  | 1945-49 | 68 | 0.351 | 0.316 | 0.386 | 0.386 | 0.350 | 0.421 |  |
| 3 | 1940-44 | 62 | 0.399 | 0.365 | 0.433 | 0.416 | 0.383 | 0.450 |  |
|  | 1940-44 | 63 | 0.388 | 0.357 | 0.418 | 0.405 | 0.375 | 0.435 |  |
|  | 1940-44 | 64 | 0.377 | 0.349 | 0.405 | 0.394 | 0.366 | 0.422 |  |
|  | 1940-44 | 65 | 0.368 | 0.341 | 0.395 | 0.384 | 0.357 | 0.411 |  |
|  | 1940-44 | 69 | 0.337 | 0.310 | 0.364 | 0.351 | 0.323 | 0.379 |  |
|  | 1940-44 | 70 | 0.331 | 0.303 | 0.360 | 0.345 | 0.315 | 0.374 |  |
|  | 1940-44 | 71 | 0.326 | 0.296 | 0.356 | 0.339 | 0.308 | 0.370 |  |
|  | 1940-44 | 73 | 0.318 | 0.282 | 0.354 | 0.330 | 0.294 | 0.366 |  |
| 4 | 1935-39 | 67 | 0.411 | 0.375 | 0.448 | 0.394 | 0.359 | 0.430 |  |
|  | 1935-39 | 68 | 0.396 | 0.364 | 0.428 | 0.379 | 0.347 | 0.410 |  |
|  | 1935-39 | 69 | 0.381 | 0.352 | 0.411 | 0.364 | 0.335 | 0.394 |  |
|  | 1935-39 | 70 | 0.368 | 0.339 | 0.396 | 0.351 | 0.323 | 0.379 |  |
|  | 1935-39 | 74 | 0.323 | 0.293 | 0.352 | 0.306 | 0.277 | 0.335 |  |
|  | 1935-39 | 75 | 0.314 | 0.283 | 0.344 | 0.298 | 0.268 | 0.328 |  |
|  | 1935-39 | 76 | 0.306 | 0.274 | 0.338 | 0.290 | 0.259 | 0.321 |  |
|  | 1935-39 | 78 | 0.292 | 0.255 | 0.330 | 0.277 | 0.241 | 0.313 |  |
| 5 | 1930-34 | 72 | 0.318 | 0.278 | 0.359 | 0.295 | 0.257 | 0.334 |  |
|  | 1930-34 | 73 | 0.307 | 0.270 | 0.344 | 0.285 | 0.250 | 0.319 |  |
|  | 1930-34 | 74 | 0.297 | 0.262 | 0.332 | 0.275 | 0.243 | 0.306 |  |
|  | 1930-34 | 75 | 0.288 | 0.254 | 0.322 | 0.266 | 0.236 | 0.296 |  |
|  | 1930-34 | 79 | 0.260 | 0.226 | 0.294 | 0.239 | 0.210 | 0.269 |  |
|  | 1930-34 | 80 | 0.255 | 0.220 | 0.290 | 0.235 | 0.204 | 0.266 |  |
|  | 1930-34 | 81 | 0.251 | 0.214 | 0.288 | 0.231 | 0.199 | 0.263 |  |
|  | 1930-34 | 83 | 0.245 | 0.203 | 0.287 | 0.226 | 0.189 | 0.263 |  |
| 6 | 1900-29 | 80 | 0.293 | 0.254 | 0.333 | 0.217 | 0.188 | 0.247 |  |
|  | 1900-29 | 81 | 0.279 | 0.243 | 0.316 | 0.206 | 0.180 | 0.231 |  |
|  | 1900-29 | 82 | 0.267 | 0.233 | 0.300 | 0.195 | 0.171 | 0.219 |  |
|  | 1900-29 | 83 | 0.255 | 0.222 | 0.288 | 0.186 | 0.163 | 0.208 |  |
|  | 1900-29 | 86 | 0.219 | 0.185 | 0.254 | 0.159 | 0.135 | 0.182 |  |
|  | 1900-29 | 87 | 0.213 | 0.177 | 0.249 | 0.154 | 0.129 | 0.179 |  |
|  | 1900-29 | 88 | 0.207 | 0.169 | 0.245 | 0.150 | 0.124 | 0.177 |  |
|  | 1900-29 | 89 | 0.199 | 0.156 | 0.241 | 0.145 | 0.114 | 0.176 |  |

| Southern care regime | | | | | | | | |
| --- | --- | --- | --- | --- | --- | --- | --- | --- |
|  |  |  | Men | | | Women | | |
| Cohort | Birth years | Age | PP | LCI | UCI | PP | LCI | UCI |
| 1 | 1950-54 | 52 | 0.273 | 0.222 | 0.325 | 0.365 | 0.322 | 0.408 |
|  | 1950-54 | 53 | 0.285 | 0.237 | 0.334 | 0.379 | 0.339 | 0.419 |
|  | 1950-54 | 54 | 0.293 | 0.247 | 0.340 | 0.389 | 0.351 | 0.427 |
|  | 1950-54 | 55 | 0.297 | 0.252 | 0.342 | 0.395 | 0.357 | 0.432 |
|  | 1950-54 | 59 | 0.274 | 0.234 | 0.314 | 0.369 | 0.331 | 0.407 |
|  | 1950-54 | 60 | 0.260 | 0.221 | 0.299 | 0.353 | 0.314 | 0.391 |
|  | 1950-54 | 61 | 0.243 | 0.205 | 0.281 | 0.333 | 0.294 | 0.371 |
|  | 1950-54 | 63 | 0.203 | 0.166 | 0.241 | 0.284 | 0.244 | 0.325 |
| 2 | 1945-49 | 57 | 0.251 | 0.208 | 0.294 | 0.340 | 0.301 | 0.378 |
|  | 1945-49 | 58 | 0.262 | 0.222 | 0.302 | 0.353 | 0.318 | 0.389 |
|  | 1945-49 | 59 | 0.269 | 0.231 | 0.307 | 0.363 | 0.330 | 0.396 |
|  | 1945-49 | 60 | 0.272 | 0.236 | 0.309 | 0.367 | 0.336 | 0.399 |
|  | 1945-49 | 64 | 0.249 | 0.217 | 0.281 | 0.342 | 0.312 | 0.372 |
|  | 1945-49 | 65 | 0.236 | 0.205 | 0.267 | 0.325 | 0.295 | 0.355 |
|  | 1945-49 | 66 | 0.220 | 0.189 | 0.250 | 0.306 | 0.275 | 0.336 |
|  | 1945-49 | 68 | 0.182 | 0.152 | 0.212 | 0.259 | 0.227 | 0.292 |
| 3 | 1940-44 | 62 | 0.265 | 0.228 | 0.303 | 0.340 | 0.301 | 0.380 |
|  | 1940-44 | 63 | 0.270 | 0.235 | 0.304 | 0.346 | 0.310 | 0.383 |
|  | 1940-44 | 64 | 0.270 | 0.238 | 0.302 | 0.347 | 0.313 | 0.382 |
|  | 1940-44 | 65 | 0.266 | 0.236 | 0.297 | 0.344 | 0.311 | 0.378 |
|  | 1940-44 | 69 | 0.219 | 0.191 | 0.247 | 0.291 | 0.260 | 0.323 |
|  | 1940-44 | 70 | 0.201 | 0.174 | 0.228 | 0.270 | 0.240 | 0.301 |
|  | 1940-44 | 71 | 0.182 | 0.156 | 0.208 | 0.247 | 0.217 | 0.277 |
|  | 1940-44 | 73 | 0.142 | 0.116 | 0.167 | 0.197 | 0.167 | 0.227 |
| 4 | 1935-39 | 67 | 0.189 | 0.157 | 0.222 | 0.247 | 0.213 | 0.281 |
|  | 1935-39 | 68 | 0.200 | 0.169 | 0.231 | 0.260 | 0.228 | 0.292 |
|  | 1935-39 | 69 | 0.207 | 0.177 | 0.237 | 0.270 | 0.240 | 0.300 |
|  | 1935-39 | 70 | 0.212 | 0.183 | 0.241 | 0.276 | 0.246 | 0.305 |
|  | 1935-39 | 74 | 0.198 | 0.170 | 0.227 | 0.262 | 0.232 | 0.291 |
|  | 1935-39 | 75 | 0.188 | 0.160 | 0.216 | 0.249 | 0.219 | 0.280 |
|  | 1935-39 | 76 | 0.176 | 0.147 | 0.204 | 0.235 | 0.204 | 0.265 |
|  | 1935-39 | 78 | 0.147 | 0.118 | 0.175 | 0.199 | 0.167 | 0.231 |
| 5 | 1930-34 | 72 | 0.190 | 0.156 | 0.225 | 0.225 | 0.181 | 0.269 |
|  | 1930-34 | 73 | 0.201 | 0.169 | 0.233 | 0.238 | 0.197 | 0.279 |
|  | 1930-34 | 74 | 0.209 | 0.178 | 0.240 | 0.247 | 0.209 | 0.286 |
|  | 1930-34 | 75 | 0.214 | 0.184 | 0.244 | 0.254 | 0.217 | 0.290 |
|  | 1930-34 | 79 | 0.201 | 0.171 | 0.232 | 0.242 | 0.211 | 0.274 |
|  | 1930-34 | 80 | 0.191 | 0.160 | 0.223 | 0.231 | 0.200 | 0.263 |
|  | 1930-34 | 81 | 0.179 | 0.147 | 0.211 | 0.218 | 0.186 | 0.250 |
|  | 1930-34 | 83 | 0.150 | 0.117 | 0.183 | 0.185 | 0.151 | 0.219 |
| 6 | 1900-29 | 80 | 0.161 | 0.128 | 0.195 | 0.155 | 0.120 | 0.190 |
|  | 1900-29 | 81 | 0.173 | 0.141 | 0.205 | 0.167 | 0.133 | 0.200 |
|  | 1900-29 | 82 | 0.182 | 0.151 | 0.213 | 0.176 | 0.144 | 0.209 |
|  | 1900-29 | 83 | 0.188 | 0.157 | 0.219 | 0.184 | 0.152 | 0.215 |
|  | 1900-29 | 86 | 0.185 | 0.151 | 0.219 | 0.184 | 0.151 | 0.217 |
|  | 1900-29 | 87 | 0.177 | 0.142 | 0.213 | 0.177 | 0.143 | 0.211 |
|  | 1900-29 | 88 | 0.168 | 0.130 | 0.205 | 0.168 | 0.133 | 0.203 |
|  | 1900-29 | 89 | 0.143 | 0.104 | 0.183 | 0.145 | 0.107 | 0.182 |

| Nordic care regime | | | | | | | | |
| --- | --- | --- | --- | --- | --- | --- | --- | --- |
|  |  |  | Men | | | Women | | |
| Cohort | Birth years | Age | PP | LCI | UCI | PP | LCI | UCI |
| 1 | 1950-54 | 52 | 0.596 | 0.549 | 0.643 | 0.609 | 0.564 | 0.653 |
|  | 1950-54 | 53 | 0.590 | 0.546 | 0.634 | 0.601 | 0.560 | 0.642 |
|  | 1950-54 | 54 | 0.582 | 0.541 | 0.623 | 0.592 | 0.553 | 0.630 |
|  | 1950-54 | 55 | 0.573 | 0.533 | 0.613 | 0.581 | 0.545 | 0.618 |
|  | 1950-54 | 59 | 0.527 | 0.488 | 0.565 | 0.529 | 0.495 | 0.564 |
|  | 1950-54 | 60 | 0.513 | 0.474 | 0.552 | 0.515 | 0.480 | 0.549 |
|  | 1950-54 | 61 | 0.499 | 0.460 | 0.538 | 0.499 | 0.464 | 0.535 |
|  | 1950-54 | 63 | 0.470 | 0.427 | 0.513 | 0.468 | 0.428 | 0.508 |
| 2 | 1945-49 | 57 | 0.568 | 0.522 | 0.613 | 0.566 | 0.526 | 0.607 |
|  | 1945-49 | 58 | 0.565 | 0.523 | 0.607 | 0.562 | 0.525 | 0.598 |
|  | 1945-49 | 59 | 0.560 | 0.521 | 0.600 | 0.556 | 0.522 | 0.590 |
|  | 1945-49 | 60 | 0.555 | 0.517 | 0.593 | 0.549 | 0.516 | 0.582 |
|  | 1945-49 | 64 | 0.522 | 0.486 | 0.558 | 0.512 | 0.480 | 0.544 |
|  | 1945-49 | 65 | 0.512 | 0.476 | 0.548 | 0.501 | 0.468 | 0.533 |
|  | 1945-49 | 66 | 0.501 | 0.464 | 0.539 | 0.489 | 0.455 | 0.523 |
|  | 1945-49 | 68 | 0.478 | 0.436 | 0.520 | 0.464 | 0.425 | 0.503 |
| 3 | 1940-44 | 62 | 0.493 | 0.450 | 0.537 | 0.488 | 0.447 | 0.529 |
|  | 1940-44 | 63 | 0.491 | 0.451 | 0.531 | 0.484 | 0.447 | 0.522 |
|  | 1940-44 | 64 | 0.488 | 0.450 | 0.525 | 0.480 | 0.444 | 0.515 |
|  | 1940-44 | 65 | 0.483 | 0.447 | 0.519 | 0.474 | 0.440 | 0.508 |
|  | 1940-44 | 69 | 0.457 | 0.420 | 0.493 | 0.443 | 0.409 | 0.477 |
|  | 1940-44 | 70 | 0.448 | 0.411 | 0.486 | 0.434 | 0.399 | 0.468 |
|  | 1940-44 | 71 | 0.439 | 0.401 | 0.478 | 0.424 | 0.388 | 0.460 |
|  | 1940-44 | 73 | 0.421 | 0.377 | 0.464 | 0.404 | 0.363 | 0.444 |
| 4 | 1935-39 | 67 | 0.436 | 0.389 | 0.482 | 0.420 | 0.372 | 0.468 |
|  | 1935-39 | 68 | 0.431 | 0.388 | 0.474 | 0.414 | 0.370 | 0.457 |
|  | 1935-39 | 69 | 0.425 | 0.385 | 0.465 | 0.407 | 0.366 | 0.448 |
|  | 1935-39 | 70 | 0.419 | 0.380 | 0.458 | 0.400 | 0.360 | 0.439 |
|  | 1935-39 | 74 | 0.387 | 0.348 | 0.426 | 0.365 | 0.327 | 0.403 |
|  | 1935-39 | 75 | 0.378 | 0.339 | 0.418 | 0.355 | 0.316 | 0.394 |
|  | 1935-39 | 76 | 0.369 | 0.328 | 0.410 | 0.345 | 0.306 | 0.385 |
|  | 1935-39 | 78 | 0.350 | 0.304 | 0.396 | 0.326 | 0.282 | 0.370 |
| 5 | 1930-34 | 72 | 0.435 | 0.379 | 0.491 | 0.397 | 0.343 | 0.450 |
|  | 1930-34 | 73 | 0.423 | 0.372 | 0.475 | 0.384 | 0.335 | 0.433 |
|  | 1930-34 | 74 | 0.411 | 0.363 | 0.459 | 0.371 | 0.325 | 0.416 |
|  | 1930-34 | 75 | 0.398 | 0.351 | 0.444 | 0.357 | 0.313 | 0.401 |
|  | 1930-34 | 79 | 0.343 | 0.298 | 0.387 | 0.302 | 0.260 | 0.344 |
|  | 1930-34 | 80 | 0.329 | 0.284 | 0.374 | 0.289 | 0.246 | 0.331 |
|  | 1930-34 | 81 | 0.315 | 0.269 | 0.361 | 0.276 | 0.232 | 0.319 |
|  | 1930-34 | 83 | 0.289 | 0.239 | 0.339 | 0.251 | 0.205 | 0.297 |
| 6 | 1900-29 | 80 | 0.290 | 0.247 | 0.333 | 0.234 | 0.196 | 0.272 |
|  | 1900-29 | 81 | 0.280 | 0.241 | 0.319 | 0.224 | 0.190 | 0.258 |
|  | 1900-29 | 82 | 0.270 | 0.234 | 0.306 | 0.215 | 0.183 | 0.246 |
|  | 1900-29 | 83 | 0.260 | 0.225 | 0.295 | 0.205 | 0.175 | 0.235 |
|  | 1900-29 | 86 | 0.222 | 0.185 | 0.258 | 0.171 | 0.141 | 0.202 |
|  | 1900-29 | 87 | 0.213 | 0.174 | 0.251 | 0.164 | 0.132 | 0.196 |
|  | 1900-29 | 88 | 0.204 | 0.164 | 0.244 | 0.157 | 0.123 | 0.190 |
|  | 1900-29 | 89 | 0.187 | 0.143 | 0.232 | 0.143 | 0.106 | 0.180 |
