# Supplementary material for: Cohort Trajectories by Age and Gender for Informal Caregiving in Europe Adjusted for Sociodemographic Changes, 2004 and 2015
Source: J Gerontol B Psychol Sci Soc Sci. 2023 Jan 23;78(8):1412–22. doi: 10.1093/geronb/gbad011 (PMC10394995; doi:10.1093/geronb/gbad011)
Supplement: gbad011_suppl_Supplementary_Material_S3 [file gbad011_suppl_supplementary_material_s3.docx]

**Supplementary Material 3:** Individual response rates (in percentage) by country at baseline (wave 1)

| Country | Individual response rate (upper bound estimate) |
| --- | --- |
| Austria | 45.3 |
| France | 92.7 |
| Germany | 50.3 |
| Netherlands | 54.1 |
| Switzerland | 38.3 |
| Belgium | 36.4 |
| Spain | 37.9 |
| Italy | 43.7 |
| Greece | 63.6 |
| Denmark | 62.5 |
| Sweden | 45.5 |
| Total | 52.9 |

Source: Adapted from Bergman et al. (2019).
